# A synthetic urease-mimetic catalyst for wind erosion control via carbonate precipitation

**DOI:** 10.1038/s41598-026-52936-3

**Published:** 2026-05-14

**Authors:** Alireza Dehghani, Ghassem Habibagahi, Fatemeh Pakpour, Elham Safaei, Ehsan Nikooee

**Affiliations:** 1 Department of Civil and Environmental Engineering, School of Engineering, Shiraz University, Shiraz, Iran; 2 Department of Chemistry, College of Sciences, Shiraz University, Shiraz, Iran

**Keywords:** Wind erosion, Urease-mimetic catalyst, Schiff-base complexes, Wind tunnel, Sand dust, Biogeochemistry, Environmental sciences

## Abstract

Wind erosion in arid and semi-arid regions poses a significant global environmental challenge, threatening infrastructure and human health. Conventional enzymatic-induced carbonate precipitation (EICP) for soil stabilization, while effective, relies on the natural urease enzyme extracted from plants or microorganisms, thereby being limited by extraction methods, purification levels, and the availability of biological sources, as well as the natural enzyme’s environmental sensitivity. This study introduces a novel approach utilizing synthetic urease-mimetic catalysts to induce calcite precipitation for dust suppression. Two Schiff base complexes, derived from glycine and salicylaldehyde with central metal ions of copper(II) and zinc(II), were synthesized and evaluated as catalysts for urea hydrolysis. The zinc-based complex was selected as the superior catalyst based on higher calcite precipitation yield and lower cost. Through response surface methodology (RSM) and wind tunnel testing on erodible siliceous sand, the optimal application parameters were determined to be a solution containing 8 g/L urea, 13 g/L CaCl₂, and 1 g/L Zn-complex applied at a rate of 1.65 L/m². This treatment effectively reduced wind erosion to negligible levels (mass loss per original mass of the sample ~ 1%). Supplementary tests, including successive wet-dry cycles, surface strength measurement, and SEM analysis, confirmed the formation of a durable calcite crust that bonds soil particles, increasing surface strength to over 150 kPa. The stabilized soil exhibited excellent resistance to simulated environmental stressors, including heat (50 °C) and long-term aging (one year), with only a minimal increase in erosion (mass loss percent of 1.5%). This research demonstrates that Schiff base complexes, particularly the zinc variant, are highly effective and durable catalysts for soil stabilization via chemical carbonate precipitation, offering a promising alternative to biological enzyme-based methods.

**Supplementary Information:**

The online version contains supplementary material available at 10.1038/s41598-026-52936-3.

## Introduction

Globally, natural hazards such as volcanic eruptions, earthquakes, droughts, floods, and storms represent significant threats to the natural environment, ecosystems, and human societies, causing extensive damage, including environmental degradation, habitat destruction, and loss of life. Among these phenomena, dust storms also inflict substantial damage on human communities and infrastructure. While occurring in various global regions, statistical data indicate that arid and semi-arid areas are particularly susceptible to this phenomenon^1^. Wind erosion is a major challenge to achieving sustainable agricultural development, largely exacerbated by human-induced climate change, and is considered the third most significant global environmental challenge^2^.

Primary global regions affected by wind erosion include the Sahara Desert in North Africa^3–5^, the Thar Desert^6–8^, the Altiplano region encompassing Peru, Bolivia, and Chile in South America^9^, and the sandy deserts of western and northwestern China, which are major dust sources in Asia^10^. In North America, wind erosion occurs predominantly in the Great Plains due to high wind velocities and insufficient vegetation cover^10^. Australia has experienced increased vulnerability to wind erosion due to expanded livestock grazing and the consequent loss of vegetative cover in agricultural zones^10,11^. In the Middle East, the most severe wind erosion occurs in the alluvial plains of Iraq, Kuwait, and Iran, which cover vast arid and semi-arid areas and are primarily driven by northwesterly winds during the summer months^10^.

Various methodologies exist for stabilizing soils susceptible to dust phenomena. These include biological stabilization using enzymes, physico-mechanical stabilization^12^, chemical stabilization^13,14^, and novel approaches employing new materials^15^. The primary objective of these methods is to enhance soil properties and increase surface resistance to wind erosion. Most techniques seek an effective additive that can induce uniform cohesion among soil particles, withstand environmental factors such as wind and rain, demonstrate high durability, and be environmentally benign and non-toxic to humans. Promising techniques include the application of mulch and cementitious materials for dust source stabilization, the use of microorganisms, and the application of biopolymers^2,16^. Biopolymers such as chitosan, guar gum, and xanthan gum have been investigated for soil erosion control with favorable results^16^.

A critical natural cementing agent is calcium carbonate (calcite), which constitutes approximately 4% of the Earth’s crust and is the most abundant natural cement^17,18^. In nature, calcite precipitation in soils, a process that typically takes hundreds of years, can be accelerated microbially (Microbially Induced Carbonate Precipitation, MICP) through the hydrolysis of urea by the enzyme urease^19,20^. This process has been studied in recent years, given its potential for soil stabilization, improvement of problematic soils and dust suppression^8,21–29^. In this method, hydrolysis of urea produces carbonate ions, which facilitate the precipitation of calcite in the presence of calcium ions (e.g., from calcium chloride)^30–32^. While MICP using bacteria like *Sporosarcina pasteurii* is well-studied^33,34^, it faces challenges including complex bacterial activity control, susceptibility to non-sterile conditions, and inhibition factors like pH, salt concentration, and temperature^35,36^. An alternative is to use the urease enzyme directly^37^. Enzyme-Induced Carbonate Precipitation (EICP) is a bio-inspired ground improvement technique. Studies have demonstrated that EICP can effectively stabilize soil, significantly increasing its resistance to wind erosion and unconfined compressive strength^38–41^. In addition, biopolymers have also been introduced as another potential biological method for soil stabilization^1,7,16,42,43^. However, biopolymers and enzymes have to be sourced from plants, fungi or microorganisms. The need to biological sources can be alleviated through alternative techniques such as synthesizing biomimicking urease enzymes.

This study, therefore, proposes a novel approach: utilizing synthetic urease-mimetic catalysts to overcome the limitations of biological methods. Schiff base complexes, organic compounds featuring a functional group (C = N-R), can act as ligands to form stable complexes with transition metal ions^44,45^. The metal center in these complexes can mimic the active site of natural enzymes, for instance, urease^46^. Previous limited studies have shown that metal ions, such as Nickel (Ni²⁺), can catalyze urea hydrolysis rates up to 10⁴ times faster than the uncatalyzed reaction^47,48^. However, such studies have not examined the potential of the method for enzymatically-induced calcium carbonate precipitation^46–48^, and feasibility of the method for soil stabilization and dust suppression has also remained unexplored.

In this study, for the first time, Schiff base complexes are employed as urease enzyme models to induce calcite precipitation and stabilize soil. Two complexes were synthesized and characterized:

1. A Schiff base complex derived from the amino acid glycine with copper as the central metal ion. A Schiff base complex derived from the amino acid glycine with zinc as the central metal ion.
2. These metals were selected based on their reported catalytic activity and comparatively lower cost. After characterizing these two Schiff base complexes, the more effective catalyst was selected. The objectives include determining the optimal concentration and application rate of the stabilizing solution required to effectively control wind erosion in treated sand specimens through wind tunnel testing and complementary analyses.

## Materials and methods

This section first describes the synthesis and characterization of these synthetic enzymes (catalysts) and discusses their relative advantages. It then describes the soil stabilization procedure employing an optimized catalyst-to-reactants ratio, followed by wind tunnel testing. As discussed earlier, in common enzymatic approach, urea hydrolysis is facilitated through urease enzyme, thereby generating carbonate ions (CO₃²⁻), which react with calcium ions (Ca²⁺) to form calcium carbonate. In this study, Schiff base complexes—serving as enzyme mimics (pseudo-enzymes)—were used to catalyze urea hydrolysis. While various metal complexes (e.g., with Ni, Co, Mn, Ru, or Pd centers) are known to catalyze this reaction, copper and zinc were selected for their comparatively lower cost. Consequently, two urease-mimetic catalysts were investigated: a glycine-derived Schiff base complex with copper as the central metal ion, along with a second complex of the same type, where zinc acts as the central metal ion. Both complexes were synthesized and characterized using the same method, differing only in the metal salt precursor.

### Preparation and synthesis of Schiff Base complexes

The synthesis of these complexes was carried out according to the reported methods to make complexes with metal ions^49,50^. Materials required for synthesizing the Schiff base complexes are detailed in Table 1. The synthesis and characterization techniques for these complexes are introduced hereafter.

**Table 1:** Details of materials required for the synthesis of Schiff base complexes.

Chemical Name	Molar Mass (g/mol)	formula
Salicylaldehyde	122.12	C_6_H_4_CHO-2-OH
Glycine	75.07	NH_2_-CH_2_‐COOH
Copper(II) acetate	181.63	Cu(CH_3_COO)_2_
Zinc(II) acetate	219.5	Zn(CH_3_COO)_2_
Ethanol	46.07	C_2_H_5_OH

#### Synthesis

**Fig. 1:**
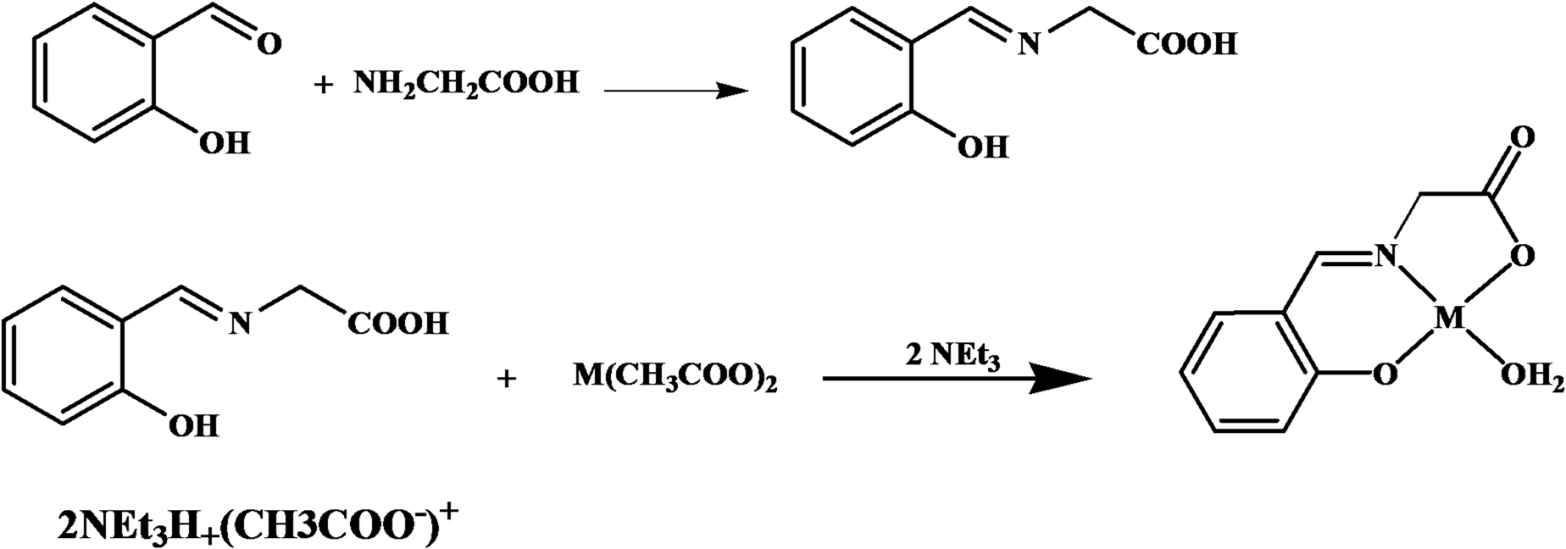
Synthetic pathway of ligand and metal complexes (M = Zn(II), Cu(II)).

To prepare the solution, 0.005 moles of salicylaldehyde (0.611 g) were dissolved in 20.0 cm^3^ of 99% C_2_H_5_OH. Then, 0.005 moles of glycine were added dropwise to 10.0 cm^3^ of ultrapure water while stirring constantly. The mixture was heated to 55˚C and kept in a water bath for approximately 4 h. This resulted in the formation of a reddish yellow colored Schiff base solution. Next, a filtered solution containing 0.005 moles of copper(II) and zinc(II) acetate in 10.0 cm^3^ of ultrapure water was added dropwise to the Schiff base solution while stirring constantly. The mixture was then kept at 55˚C for about 6 h and left to stand at room temperature for 2–3 hours. This resulted in the formation of light green and pale yellow precipitates of [Cu^II^(Gly)(Sal)(H_2_O)] and [Zn^II^(Gly)(Sal)(H_2_O)], respectively (Fig. 1).

For the synthesis of the Schiff base copper (II) and zinc complexes, first, the Schiff base ligand was synthesized via the condensation reaction of salicylaldehyde and the amino acid glycine. Subsequently, the Schiff base complex was produced by reacting the Schiff base ligand with metal acetate salts of copper(II) and zinc(II). Triethylamine base was used to deprotonate the Schiff base and facilitate its coordination to the metal ions.

#### Characterization

The produced Schiff base complexes were characterized using four different methods: Fourier Transform Infrared (FTIR) spectroscopy (Shimadzu FT-IR 8300), CHN (i.e., carbon (C), hydrogen (H), nitrogen (N)) elemental analysis (through combustion and subsequently, gas chromatography using Themo Finnigan-Flash 1200), ^1^H NMR, and single crystal X-ray crystallography (XtaLAB AFC11 (RCD3)). ^1^H NMR Spectrum was recorded on a 250 MHz Bruker DRX instrument utilizing DMSO-d6 and tetramethylsilane (TMS).

#### Optimization of catalyst and reactants concentration

The primary reactants in this study were urea and calcium chloride. Previous studies have used various ratios of urea and calcium chloride, ranging from 0.05 M to 2 M in enzymatic calcite precipitation methods^38^. Another study using Schiff base complexes for urea hydrolysis used equal concentrations of urea and the complex at 0.0001 M^46^. In this research, the optimal ratio of reactants was determined empirically based on the use of the Schiff base complex.

For the comparison purposes, different amounts of each catalyst were evaluated. Based on economic considerations and the precipitation yield of each complex during synthesis and the resulting calcite, the more suitable catalyst was selected for further experiments and solution preparation. In order to find the optimal value for catalyst, a quantitative measure suitable for measuring efficiency of synthesized urease mimicking complexes in calcium carbonate precipitation, which is the ultimate goal (namely, Prod./Cat., i.e., the ratio of produced calcium carbonate to catalyst weight) was considered and assessed to find the optimum value for catalyst. This ratio was optimized to achieve higher precipitation for unit enzyme weight thereby helping to reduce process cost. Further refining of the reactants and optimization of their values is performed based on the wind tunnel tests and their results are presented in the subsequent sections. The results of optimization procedure have been presented in Sect.  3.1.4.

### Soil sampling and characterization

For this study, areas in Fars Province, Iran, susceptible to wind erosion were selected for sampling. The Dejgah catchment in southwestern Fars is covered by soil layers that are considerably prone to wind erosion. The severity of soil erosion in this region has been discussed in previous studies such as Owji et al.^16^. Wind erosion susceptibility is commonly categorized into four degrees: severe, high, moderate, and light^51^. In this catchment, a region with known severe erosion was selected, and bulk soil samples were collected from the topsoil for further investigation. The geographical location of soil sampling is positioned at the global coordinate of N 28° 11ʹ 28ʺ and E 52° 23 ʹ 55 ʺ.

**Fig. 2:**
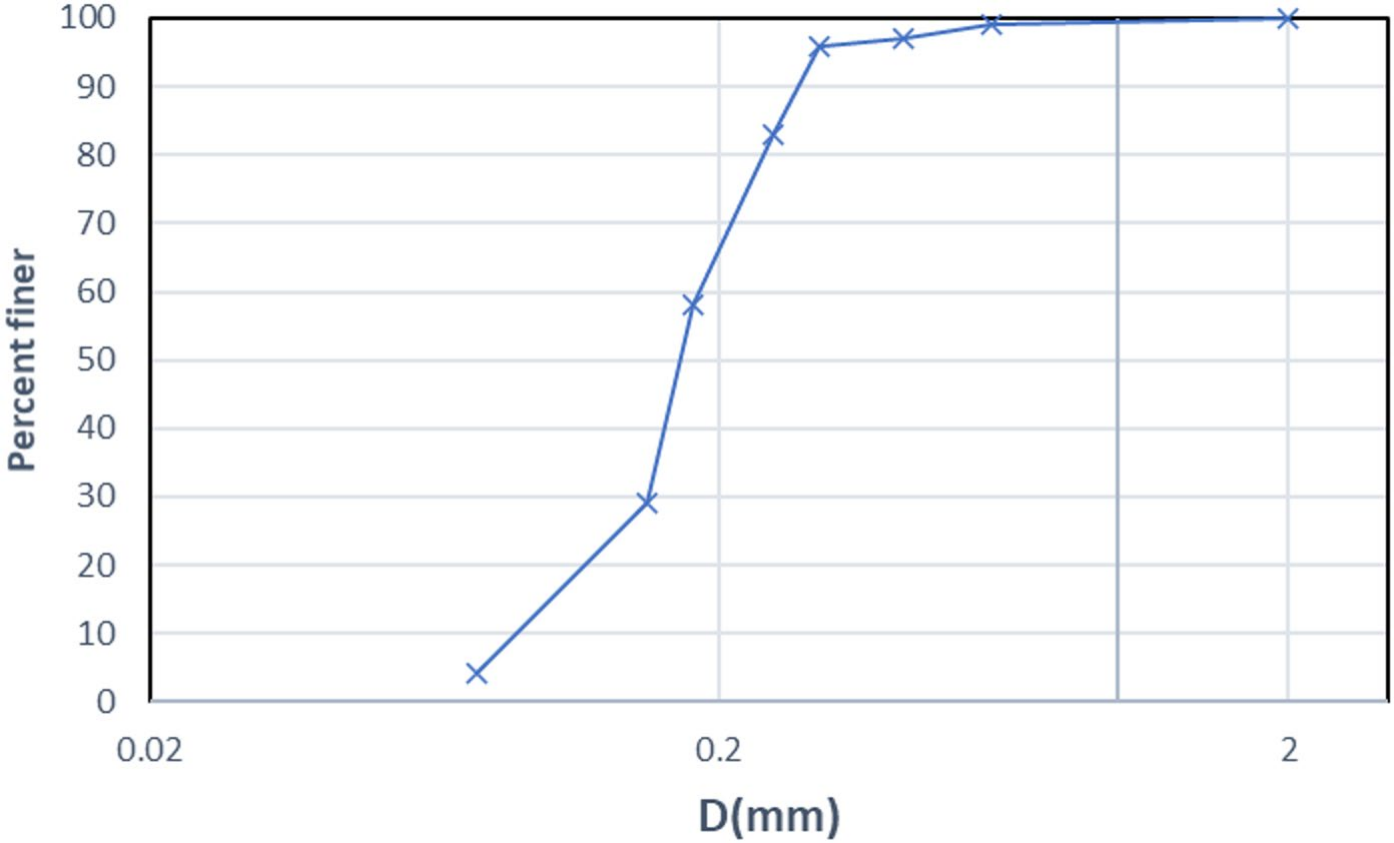
Particle size distribution curve of the soil.

Grain size distribution (sieve) analysis and Atterberg limits tests were conducted on the soil samples indicated that the soil is a poorly graded sand with 7% fines content, classified as SP-SM according to the Unified Soil Classification System (USCS). The particle size distribution of Dejgah sand is presented in Figure. 2, and basic soil indices are depicted in Table 2.

**Table 2:** Basic indices of the soil used in this study.

Index	Value
Classification (USCS)	SP-SM
P200 (%)	7
C_u_	2.02
C_c_	1.4
Max dry density (Mg/m³)	1.73
Optimum moisture content (%)	8.2

Also, Energy-Dispersive X-ray analysis (EDX) test results revealed the proportion of basic elements in the soil, as presented in Table 3. The results indicate that carbon, oxygen, and silicon are the major soil constituents, suggesting a high silicon dioxide (SiO₂) content.

**Table 3:** Results of the Energy-Dispersive X-ray analysis (EDX) analysis of the soil.

Symbol	weight% %
C	15.75
O	59.77
Na	0.76
Mg	2.35
Al	3.01
Si	12.81
K	0.44
Ca	3.86
Fe	1.29

### Sample preparation

Test container dimensions of 17 × 220 × 220 mm were selected for wind erosion tests, similar to the research by Ayeldeen et al.^52^. As shown in the grain size distribution curve, Fig. 2, more than 97% of soil grains pass this sieve number (with the opening of 0.5 mm). This sieve size was selected to screen possible few large debris from the soil sample. Completely dry soil passing through a No. 35 sieve was, therefore, placed in the container without compaction to increase surface erodibility. The soil surface was leveled uniformly using a straight edge.

Based on the concentration and application volume, two solutions—one containing urea and calcium chloride, and the other containing the catalyst—were prepared and mixed before spraying commenced. The prepared solution was sprayed onto the sample surfaces using an electric sprayer. The samples were weighed precisely and left at room temperature for 10 days to dry (i.e., until a constant weight was achieved, indicating they were dry). The sample container was then placed in the wind tunnel. The test duration, namely, 10 min, was selected to be similar to that of Owji et al.^16^. After the test was completed, the samples were weighed again to determine the amount of erosion. The erosion rate was calculated using the ratio of the weight difference of the samples before and after the wind tunnel test to the initial soil weight (Eq. 1). Finally, based on the results from sample erosion tests, the optimal concentration and application volume were selected.

1 $$\:Percent\:Erosion,\:R1=\frac{{w}_{1}-{w}_{2}}{{w}_{1}-{w}_{p}}\:\times\:100$$

where *w*_*1*_, *w*_*2*_, *and w*_*p*_ are the weight of the sample (container + soil) before the test, the weight of the sample (container + soil) after the test, and the weight of the empty container, respectively.

### Wind tunnel

**Fig. 3:**
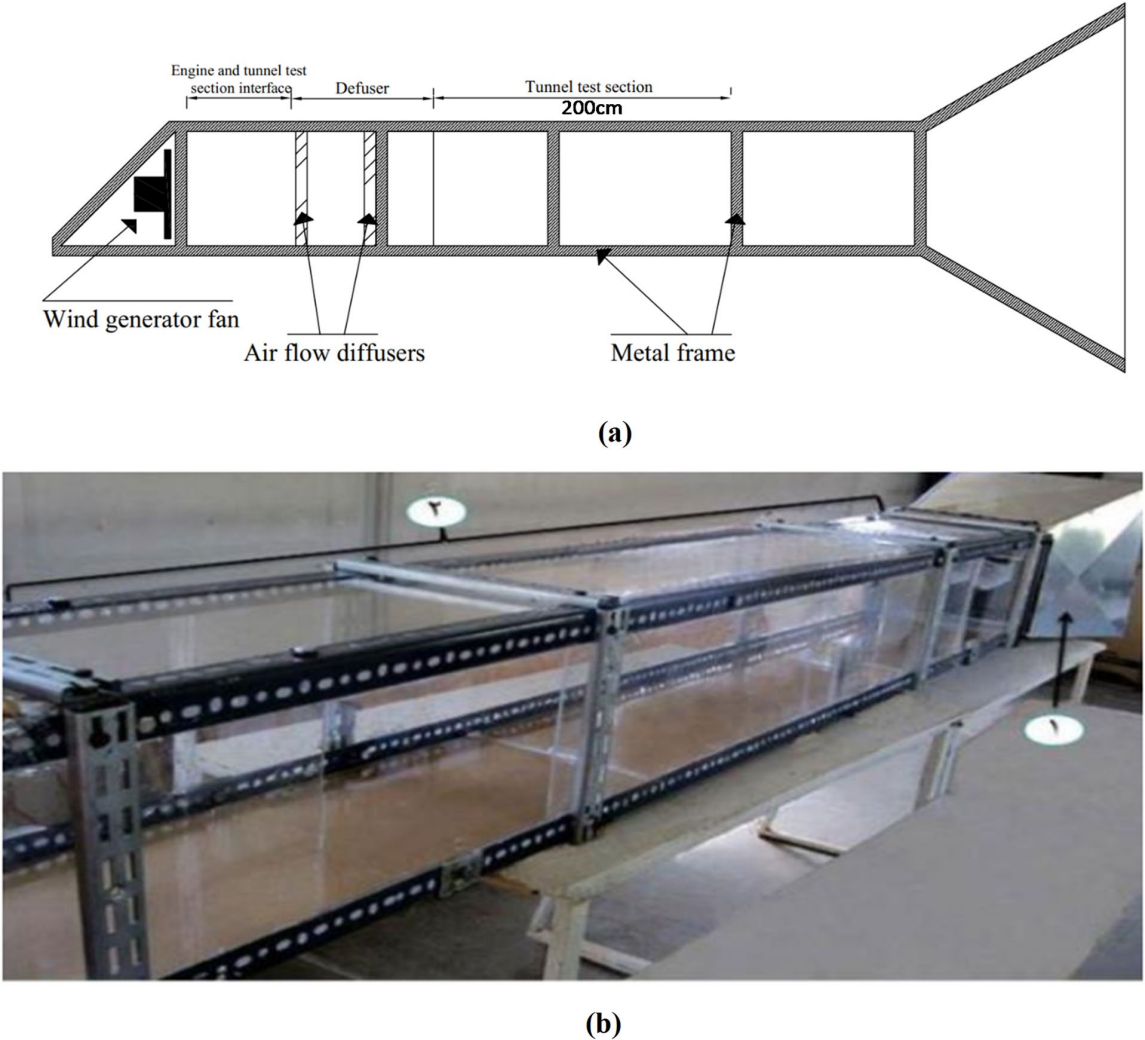
(**a**) Schematic/diagram of the wind tunnel setup. (**b**) Actual picture of wind tunnel setup.

The wind tunnel used in this research was designed and built in the Wind Erosion Laboratory of the Department of Natural Resources and Environmental Engineering at Shiraz University. The main components of this wind tunnel include a motor connector, the tunnel chamber, and a truncated pyramid. The tunnel chamber consists of two sections: the first straightens the airflow, and the second is the test section. The cross-sectional dimensions of the tunnel are 30 × 30 cm, and the length of the test section is 200 cm. A 30 × 22 cm hatch was installed in one of the Plexiglas walls for placing the sample inside the wind tunnel (Fig. 3).

**Fig. 4:**
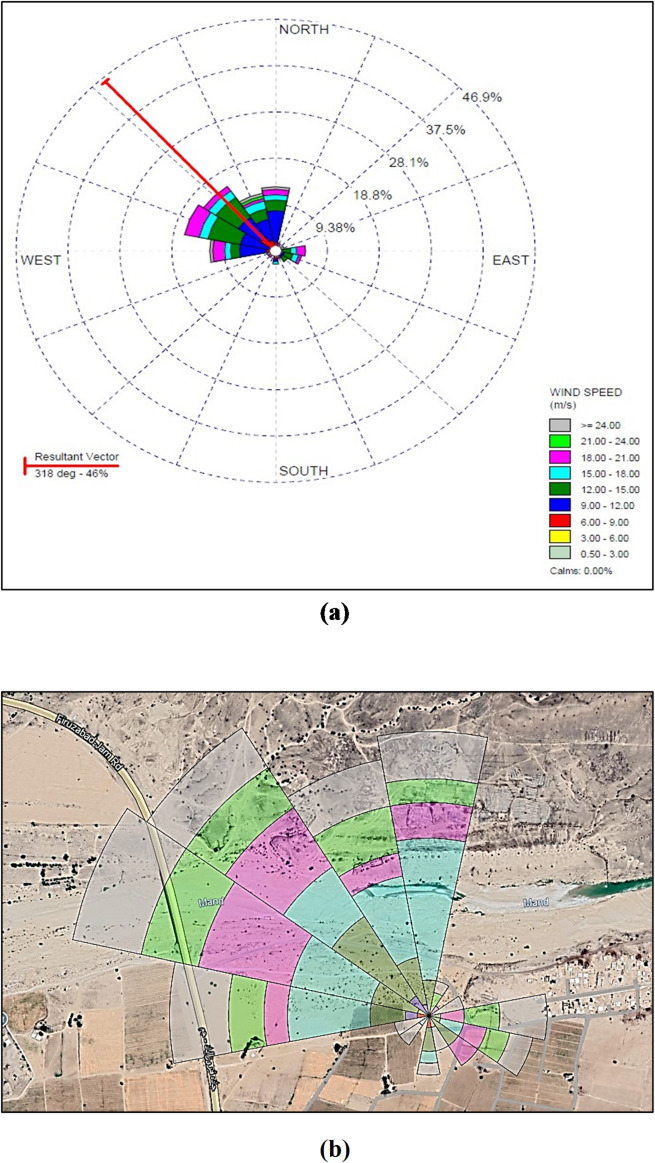
(**a**) Wind rose diagram illustrating the distribution of wind direction and speed in the region of study. (**b**) Spatial mapping of wind speed zones based on long-term observations within the investigated region (after Tourtiz et al.^53^, under CC BY license).

Figure 4 presents the distribution of wind speed and direction in the region of study. To correctly select the wind speed for wind tunnel testing, fifteen years of wind speed and direction data from the country’s Meteorological Organization, specifically from the synoptic station in the Farashband region, were obtained and used. Based on these data, the maximum wind speed in this area has been in the range 20–21.5 m/s in this period. Generally, in wind tunnel tests, maintaining stable flow much above 20–25 m/s becomes difficult due to turbulence and facility limits. Hence 20 m/s is a practical compromise between attainable lab conditions and realistic field situations and wind speed of 20 m/s was selected to achieve maximum wind speed of close relevance to the region of interest and also comparable with previous studies^38^.

### Ancillary tests (microfabric analysis, crust thickness and wind tunnel after wet-dry cycles)

Supplementary tests were conducted on the optimal sample to investigate the suitability of the proposed method further. These tests included wind tunnel tests on treated soil after successive wet-dry cycles, measurement of crust thickness, surface strength measurements, and imaging with a Scanning Electron Microscope (SEM).

#### Durability test

To assess the durability of the optimal stabilized sample, successive wet-dry cycles were performed. The procedure was as follows: after spraying the optimal stabilizing solution onto the sample, it was left at room temperature for 10 days to dry completely and allow calcite crystals to form. The sample was then tested in the wind tunnel for 10 min. Afterwards, equivalent to 1.65 L/m² of distilled water (96.8 mL in this experiment) was sprayed onto the soil surface, and the sample was placed back in the wind tunnel for another 10 min erosion test. This spray rate has been selected based on the optimization process performed in first set of wind tunnel tests, where mass loss under different solution concentrations and spray rates was measured. This cycle was repeated 10 times (Table 4), and the erosion rate was determined at each stage. Also, durability was assessed one year after stabilization by conducting a wind tunnel test on a sample treated with the optimal application volume and concentration. This sample was stored in the laboratory at an average temperature of 20 °C for 12 months.

A characteristic of arid and semi-arid regions is their high temperature. To evaluate the performance of the stabilized soil under such conditions, a 10-year record of average maximum temperatures for three cities—Ahvaz, Abadan, and Masjed Soleyman—was obtained from the Iranian Meteorological Organization^54^. The highest temperature was recorded in Abadan at 52 °C ^54–56^. To investigate the durability of the optimal sample against heat, the sample was first left at room temperature for 24 h to promote calcite crystal formation. It was then placed in an oven at 50 °C for one week. Finally, the sample’s erosion rate after 10 min in the wind tunnel was calculated.

**Table 4:**
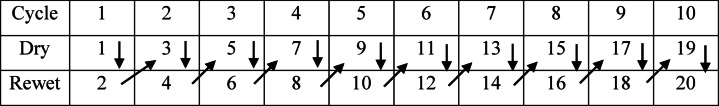
Cycle process for the wet-dry tests on the samples.

#### Crust characterization

The properties of the crust layer formed on the surface, including its thickness, surface strength and morphology were analyzed. Surface thickness was measured with a digital caliper. Surface strength was measured using a pocket penetrometer (Soiltest Inc, U.S.; Model: CL-700 A)), and following ASTM C780. Surface imaging was conducted through a Cambridge S360 Scanning Electron Microscope. All experiments were conducted in triplicate under identical conditions. Reported values represent mean results, and standard deviations are provided to demonstrate experimental repeatability.

## Results and discussion

This section first presents and analyzes the results of selecting the appropriate catalyst from the two available options, followed by an examination of the results from the wind tunnel erosion tests.

### Characterization of produced Schiff base complexes

#### FT-IR Spectral analysis

**Fig. 5:**
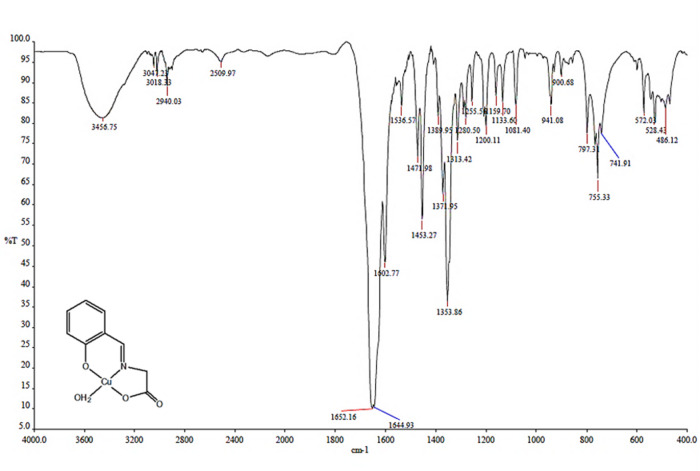
FT-IR spectrum of Cu complex.

**Fig. 6:**
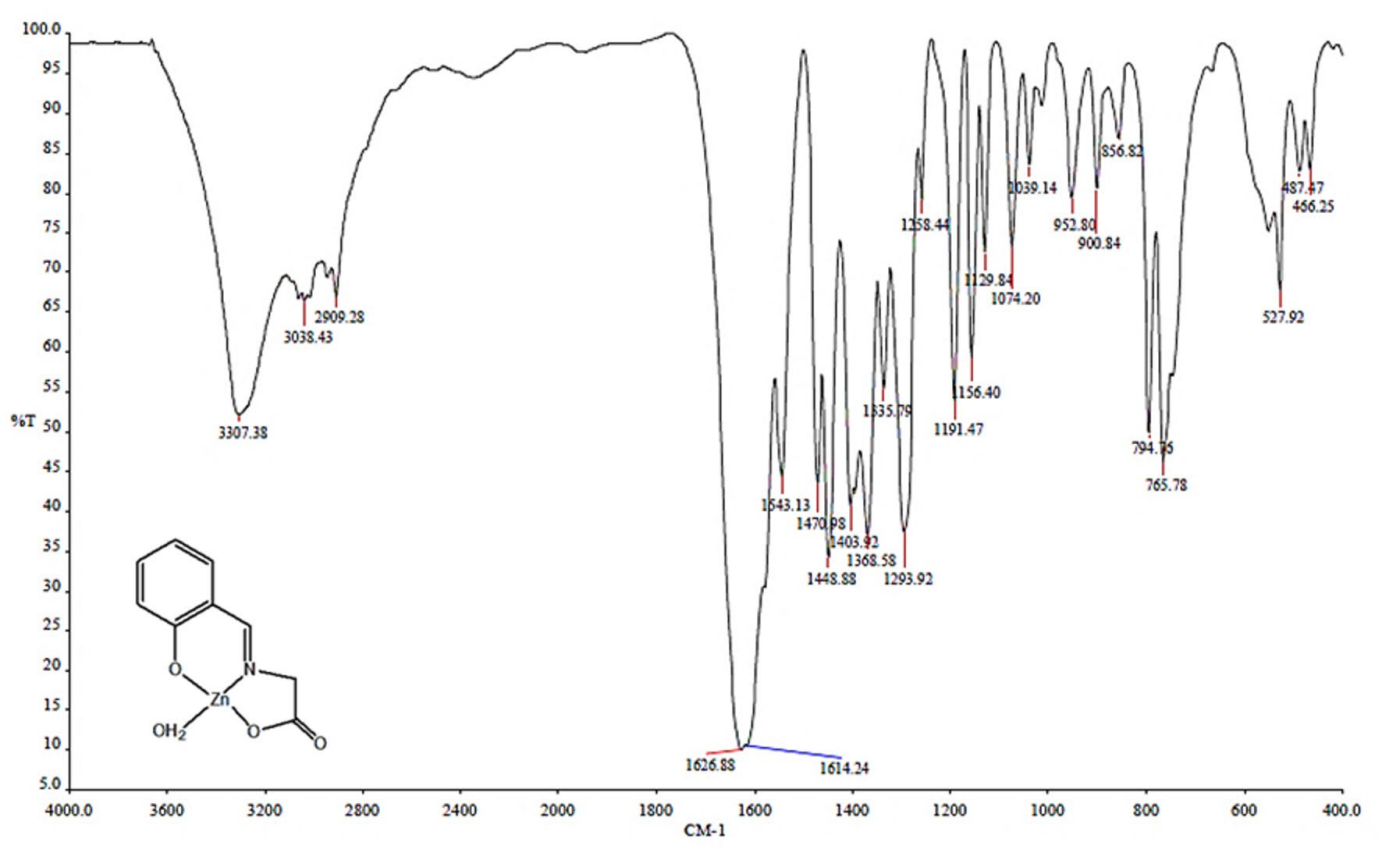
FT-IR spectrum of Zn complex.

IR spectra were recorded for the complexes in the region of 4000–400 cm^− 1^, and the observed frequencies were assigned to specific group vibrations by consulting a reference table. The FT-IR spectra of the complexes are displayed in Figs. 5 and 6.

The presence of coordinated water is indicated by the broad absorption band centered around 3300–3420 cm^− 1^. The bands at 870–900 and 730–750 cm^− 1^ may be attributed to the rocking and wagging modes of the coordinated water respectively. The IR spectrum of the Schiff base ligand shows strong bands at 1652 and 1638 cm^− 1^, which are assigned to the azomethine stretching frequency. These bands are shifted to lower frequencies (1626 –1614 cm^− 1^) during complexation, indicating that the ligand binds to the metal ions through the azomethine N atom. The phenyl group exhibits C–H stretches at 3038 cm^–1^ and C = C stretching at 1470 cm^–1^. In the spectrum of Schiff bases, a sharp band is present in the region of 3300 and 1520 cm^− 1^, which is attributed to the peptide N-H stretching frequency. However, in the complexes, this band disappears, indicating that the metal ion coordinates through the deprotonated nitrogen atom. A new broad band at 3400 cm^− 1^ can be attributed to the stretching vibration of the water molecule. Additionally, a band at 800 cm^− 1^ in the complexes is assigned to the coordinated water molecule. The Schiff base ligand also displays a band at 1368 cm^− 1^, which is due to the symmetric stretching vibration of the carboxylate group. Upon complexation, the asymmetric and symmetric stretching bands are shifted to lower frequencies, indicating the formation of a linkage between the metal ion and carboxylate oxygen atom. The spectrum of the two complexes also shows new bands in the regions of 527–535 cm^− 1^ and 466–487 cm^− 1^, which may be attributed to the formation of M-O and M-N bonds, respectively.

#### Elemental analysis

The data obtained from the elemental analysis demonstrates a strong correlation between the theoretical and experimental values. (Table 5)

**Table 5:** Elemental Analysis.

Compelex	Element	Calculated (%)	Found (%)
[Zn^II^(Gly)(Sal)(H_2_O)]	C	41.49	43.29
	H	3.48	3.39
	N	5.38	5.54
[Cu^II^(Gly)(Sal)(H_2_O)]	C	41.78	45.29
	H	3.51	2.72
	N	5.41	5.94

#### ^1^H NMR

**Fig. 7:**
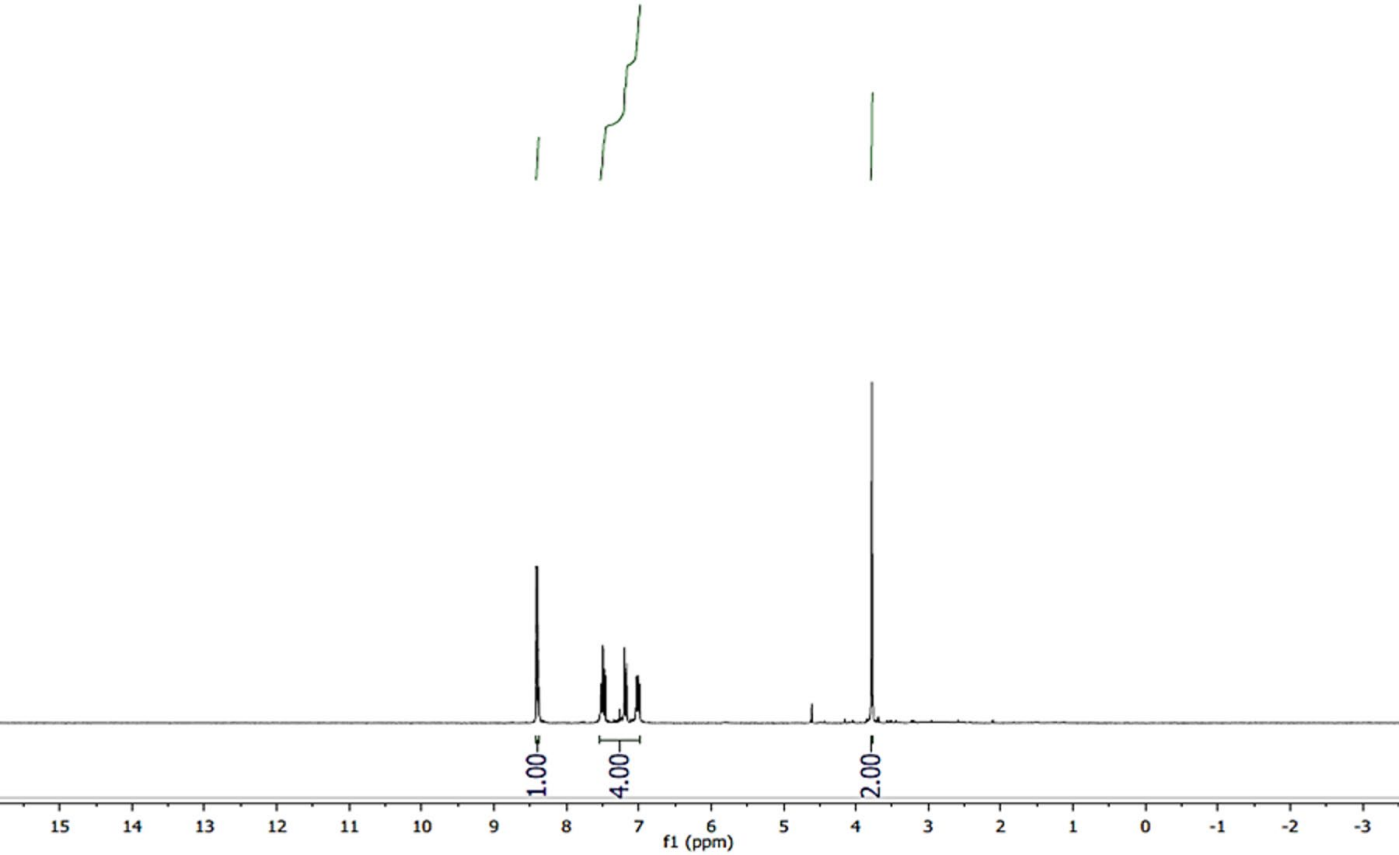
^1^H NMR spectrum of Zn complex.

Zn(II) is diamagnetic (d¹⁰), and hence it can be studied by 1H NMR; however, many specific ligands simply don’t form quality crystals or have low stability, making its crystallography difficult. The ^1^H NMR spectrum of the Zn complex in DMSO displays a singlet peak at 3.8 ppm, indicating the presence of protons in the CH_2_ group. Multiple peaks in the range of 7.0-7.6 ppm were observed for the four hydrogens in the aromatic ring. Additionally, a singlet peak at 8.3 ppm was assigned to the CH group adjacent to the nitrogen atom (Fig. 7). Cu(II) Schiff base complexes are typically paramagnetic (unpaired electrons), which severely broadens and often obliterates proton NMR signals (i.e., ^1^H peaks from ligand protons near the metal can disappear or merge into the baseline, making spectrum interpretation extremely challenging^57^, therefore, ^1^H NMR was not performed on Cu complex. Alternatively, Cu complex was characterized through crystallography. Single crystal crystallography was performed on the Cu complex, and the results are presented in the supplementary material (one may refer to Figures S1, and S2, for Cu Schiff-base complex crystal structure, and Tables S1, and S2 for its crystal data, bonds’ lengths and angles, respectively).

#### Determination of reactant quantities and catalyst selection

Various quantities of reactants were tested through a series of experiments for the two catalysts considered in this study. After conducting multiple experiments following the procedure in previous studies^46,58,59^, we were able to determine the optimal value. The optimum reactants for both catalysts were prepared by preparing a solution of 0.12 g urea and 0.19 g calcium chloride in 10 ml of distilled water. Next, a solution of the copper/zinc complex in 10 ml of distilled water was added to the reactant solution. The mixture was placed on a stirrer for 48 h for calcite precipitation. The precipitate from both solutions was weighed and presented in Table 6. Based on these results, higher amount of calcite is precipitated by the zinc(II) complex and knowing the lower cost of zinc(II) compared to copper(II), the selected catalyst for this research was the Schiff base complex with zinc as the central metal.

**Table 6:** Tests conducted to determine the optimal reactant ratio.

Test Number	Urea (g)	CaCl_2_ (g)	Catalyst (g)	Water (ml)	Precipitation (g)	Prod/Cat
1	0.012	0.019	0.0075(Cu)	4	*	*
2	0.012	0.019	0.005(Cu)	2	*	*
3	0.12	0.19	0.05(Cu)	20	0.040	0.80
4	0.12	0.19	0.025(Cu)	20	0.020	0.80
5	0. 12	0. 19	0.01(Zn)	20	0.051	5.055
6	0. 12	0. 19	0.0125(Zn)	20	0.126	10.07
7	0. 12	0. 19	0.025(Zn)	20	0.164	6.568
8	0. 12	0. 19	0.0375(Zn)	20	0.096	2.552
9	0. 12	0. 19	0.0625(Zn)	20	0.173	2.760
10	0. 12	0. 19	0.0875(Zn)	20	0.151	1.723
11	0. 12	0. 19	0.125(Zn)	20	0.189	1.512

(* undetectable precipitation).

The characterization of the precipitate resulting from the reaction was investigated using infrared spectroscopy and X-ray diffraction analysis. The results confirmed the formation of calcite.

#### Characterization of the complex-induced precipitates

**Fig. 8:**
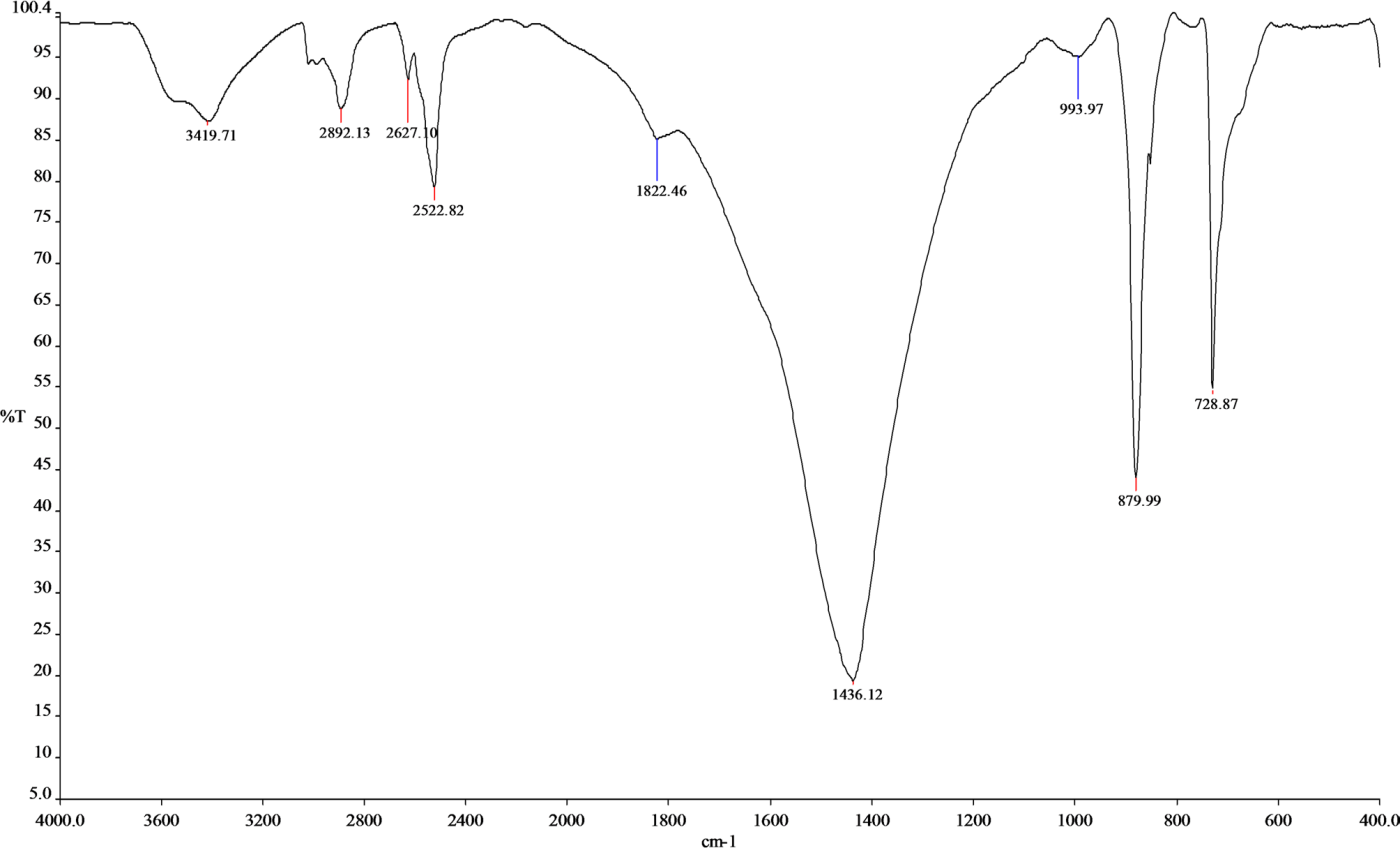
FTIR spectrum of precipitations identified as calcite.

The structure of the precipitates was investigated using Fourier transform infrared (FTIR) spectroscopy. The resulting spectrum, shown in Fig. 8, revealed absorption bands at 3419 cm^–1^, indicating the presence of O–H vibrations from H_2_O. Additionally, peaks at 728, 880, and 1436 cm^–1^ were observed, corresponding to out-of-plane bending and asymmetrical stretching vibrations of O–C–O. These IR spectra are consistent with the characteristic vibrations of calcite.

**Fig. 9:**
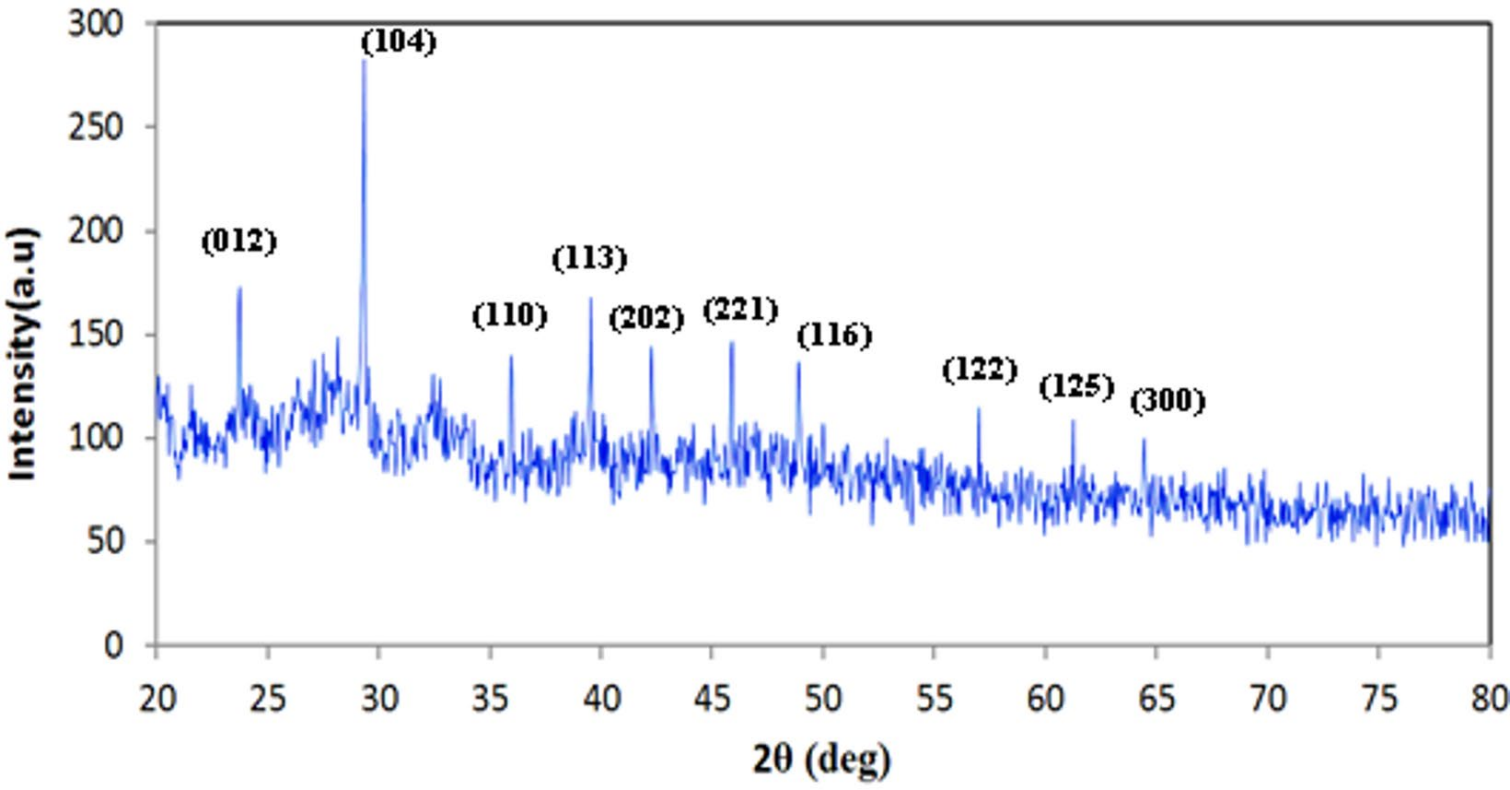
XRD spectrum of precipitations identified as calcite.

As XRD is a highly sensitive method for characterizing crystalline phases and polymorph type of inorganic compounds, further evidence from XRD was used to substantiate the formation of calcium carbonate and identify its polymorph. In Fig. 9, the X-ray diffraction pattern of the precipitated crystals are displayed. The X-ray diffraction pattern of the precipitates exhibits characteristic peaks of calcite. The characteristic peak of calcite at 2 *θ* value of 29.51 is dominant along with other peaks identified at 24.02˚, 36.08˚, 39.78˚, 43.99˚, 45.98˚, 48.99˚, 57.01˚, 63.21˚, and 64.87˚, which correspond to (hkl) indices of (104), (012), (110), (113), (202), (221), (116), (122), (125), and (300), respectively.

**Fig. 10:**
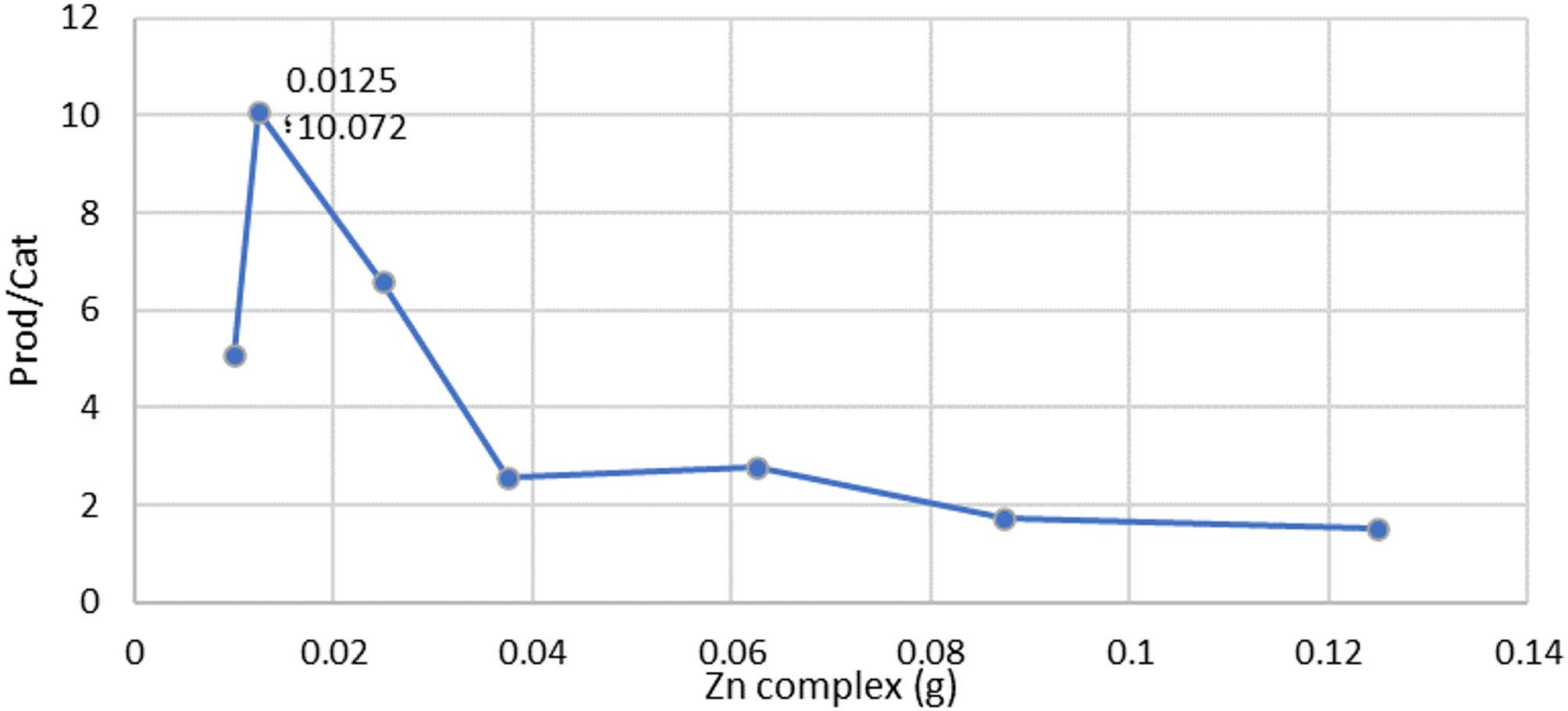
Ratio of calcite to zinc catalyst (Prod/Cat) versus catalyst mass.

Next, to determine the optimal quantity and the catalyst’s effect on calcite precipitation yield, the ratio of calcite precipitate (product) to catalyst amount was defined as the parameter **Prod/Cat**. The results of this parameter versus the amount of catalyst used are presented in Table 6 and shown in Fig. 10. As evident from Fig. 10, at a catalyst concentration of 0.0125 g in 20 mL solution (0.625 g L^− 1^), and Urea concentration of 0.1 M, the amount of calcite produced is approximately 10 times the catalyst amount, which effectively represents the optimal quantity for the zinc Schiff base complex catalyst. To have a comparison with the commercial urease enzyme, a Prod/Cat of 3.5–4.2 is estimated from tube tests of Ahenkorah et al. ^60^ for urease enzyme activities of 3.5 kU/g-40 kU/g and for the Urea concentration (0.1 M), which shows the efficiency of the proposed synthetically prepared biomimetic enzyme.

### Optimizing spray rate and reagent concentration level: wind tunnel results

Wind tunnel tests were conducted to determine the optimal concentration and spray rate required to stabilize the soil using the optimal catalyst solution. The two parameters, concentration class (C) and spray rate (V), were each divided into five levels for simultaneous optimization. All five levels of concentrations, C0.5 to C2.5 in Table 7, satisfy the optimal composition obtained previously, that is, Test No. 6 in Table 6. The effect of spray rate was investigated by varying it between 0.5 and 2.5 L/m²; these values were selected based on previous studies^1,16^. These concentration levels and spray rates were then entered into Design-Expert for experimental design, and the suggested combinations are presented in Table 8.

**Table 7:** Concentrations of reagents for each concentration class (based on the optimal catalyst quantity prepared for a 1 L solution).

Concentration Class	Urea (g)	Cacl_2_ (g)	Zn cat (g)	Water (lit)
C _0.5_	3	4.75	0.3125	1
C 1	6	9.5	0.625	1
C 1.5	9	14.25	0.9375	1
C 2	12	19	1.25	1
C 2.5	15	23.75	1.5625	1

The parameters’ level and coding by the software are shown in Table 9. As the optimal reagents resulting in maximum precipitation may not necessarily result in optimal mass loss under wind erosion, and noting that spray rate also plays a role, for optimizing both spray rate and concentration of reagents together, a concentration class has been introduced. The concentration class is a coefficient, by which all reagents’ concentrations of the base class (i.e., the one resulted in maximum precipitation per catalyst mass, determined from Table 6) are multiplied.

The high and low levels for each parameter were chosen to provide a wide range based on previous research. The results of planned experiments are also presented in Table 8. In this table, the erosion value reaches zero or a minimal amount at the central point, i.e., concentration C₁.₅ and volume V₁.₅. In Experiment 2, although concentration C₁.₅ produced good strength and cohesion, the low spray rate resulted in insufficient solution penetration into the soil, leading to significant cracking and the complete disintegration of the entire sample.

**Table 8:** Wind tunnel test results for the planned experiments.

Experiment No.	Concentration Class (C)	Spray rate (V) lit/m^2^	Erosion value %
	A	B	R1
1	C_1.5_	1.5	0
2	C_1.5_	0.5	100
3	C_1_	2	5
4	C_2_	2	0
5	C_0.5_	1.5	100
6	C_2_	1	5
7	C_1.5_	2.5	0
8	C_1_	1	98
9	C_1.5_	1.5	1
10	C_2.5_	1.5	0
11	C_1.5_	1.5	0
12	C_1.5_	1.5	3
13	C_1.5_	1.5	2

**Table 9:** Parameter levels and coding.

Variable code	Name of variable	Range of variables				
X1	Concentration Class [-]	2.5	2	1.5	1	0.5
X2	Spray rate [L/m^2^]	2.5	2	1.5	1	0.5

#### Analysis of Results

The results from the designed experiments were analyzed using the Response Surface Methodology. The influence of the two important parameters (concentration and spray volume) and their interaction effect was determined. The validity of the model was assessed by examining the normality of the residuals, the constancy of the residual variance (homoscedasticity), and the independence of the residuals.

**Fig. 11:**
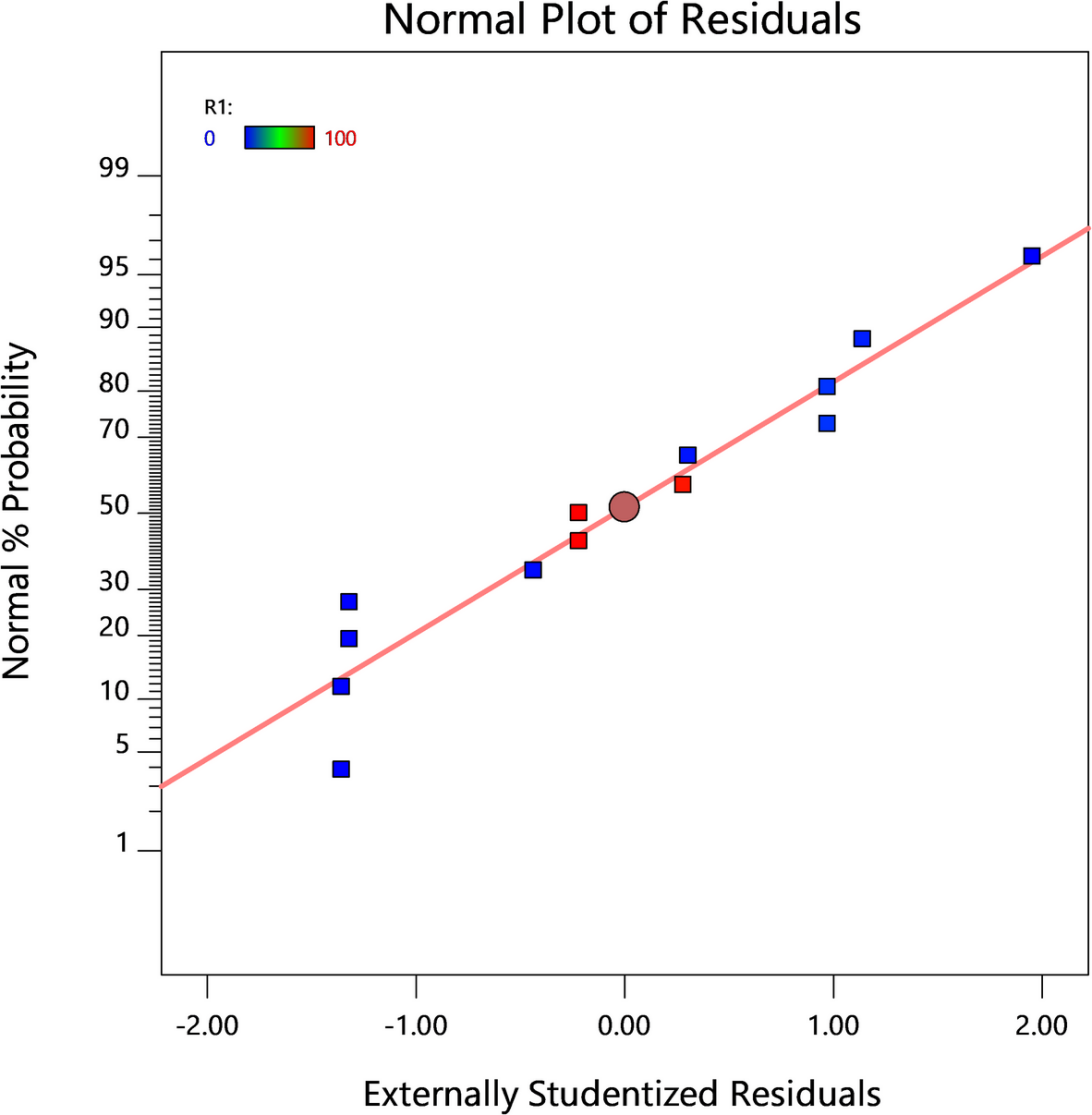
Normal plot of residuals.

A fundamental assumption in statistical data analysis is that the data follows a normal distribution. Figure 11 shows the normal probability plot of the studentized residuals. Points lying close to the straight line indicate that the data in the model are normally distributed. Deviation from this line would indicate non-normal errors.

**Fig. 12:**
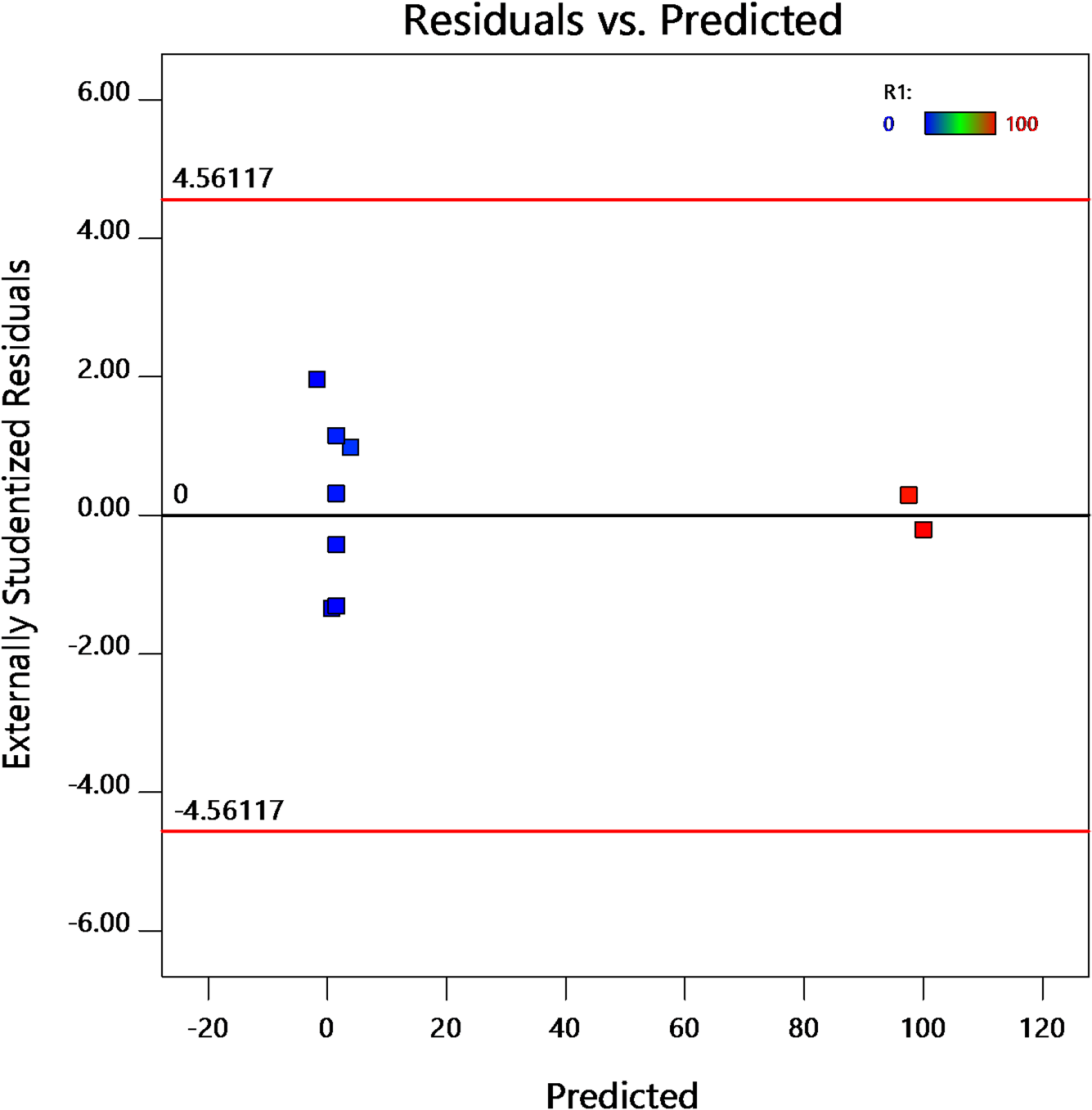
Plot of residuals versus predicted values.

The plot of residuals versus predicted values (Fig. 12) shows the points scattered randomly within a constant range. This random scatter confirms the assumption of constant variance (homoscedasticity) of the residuals. A slight funnel-shaped pattern may be observed in the residuals versus predicted plot, which is primarily attributed to the bounded nature of the response variable (wind erosion values approaching 0% and 100%) in several experimental runs.

**Fig. 13:**
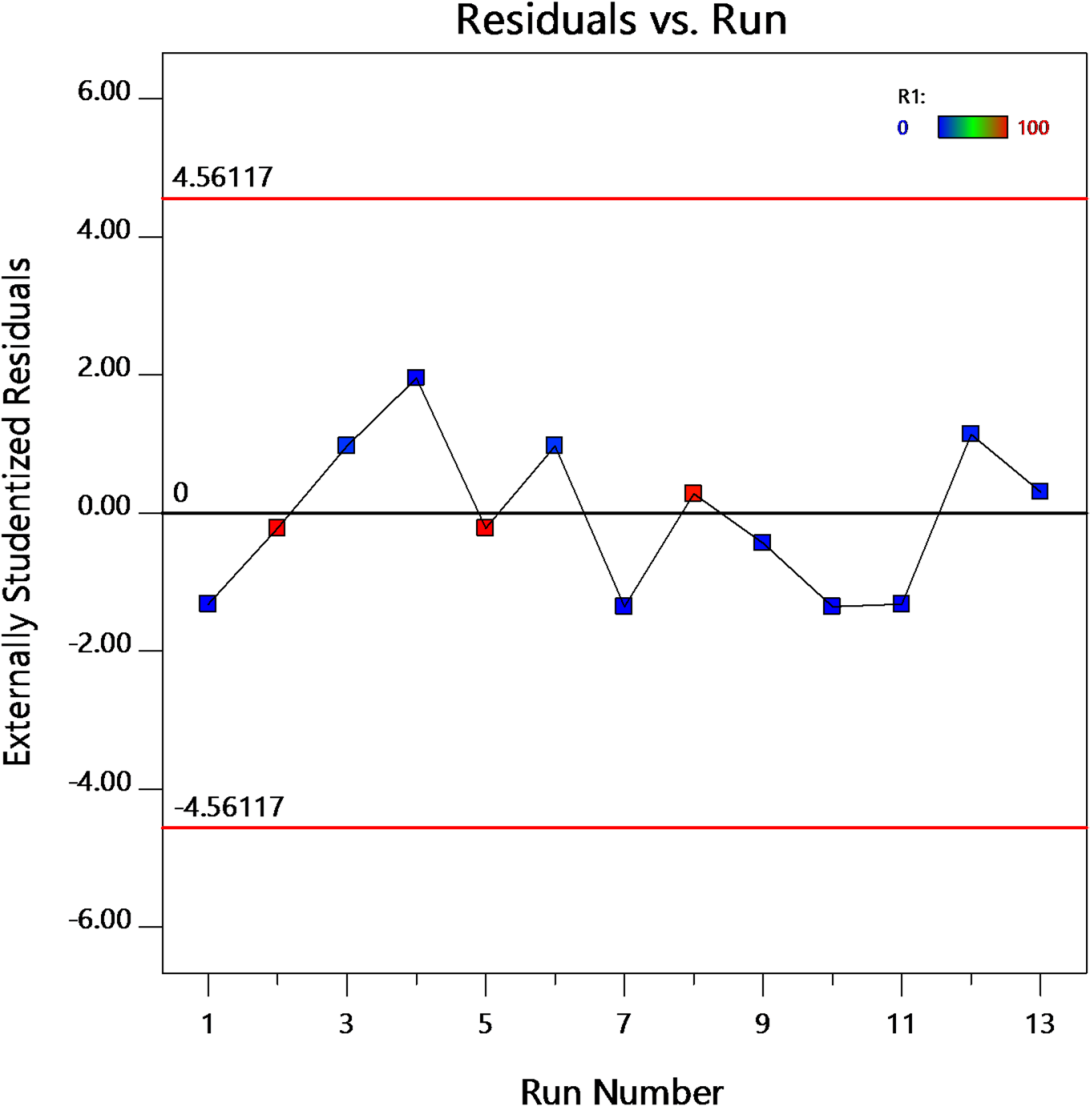
Plot of residuals versus run order.

Figure 13 shows the plot of residuals versus the run order of the experiments. This plot shows that the residuals do not exhibit a specific pattern, confirming the assumption of independence. Based on these diagnostic plots, the model is validated.

#### Model validation and statistical significance

To determine the appropriate mathematical model terms, the values of Adjusted R-squared and Predicted R-squared should be maximized, and the Lack of Fit, which compares the pure error of the design to the residual error, should be insignificant. Furthermore, if the p-value (Prob > F) for a given model term is less than 0.05, adding that term improves the selected model.

Based on the analysis shown in Table 10, the quadratic model is the best fit. It has very high values for Adjusted R-squared (0.998) and Predicted R-squared (0.997). Given the maximization of these parameters and p-values less than 0.05, the second-order (quadratic) model is recommended.

**Table 10:** Results for choosing the appropriate mathematical model.

Source	Sequential *p*-value	Lack of fit *p*-value	Adjusted *R*^2^	Predicted *R*^2^	
Linear	0.0039	< 0.0001	0.6042	0.3558	
2FI	0.1045	< 0.0001	0.6772	0.5058	
Quadratic	**< 0.0001**	**0.3842**	**0.9989**	**0.9973**	**Suggested**
Cubic	0.7717	0.1491	0.9987	0.9711	Aliased

The second-order equation for predicting erosion percentage from the coded data is given by Eq. 2. The combination of variables in the equation was selected based on significant terms with p-values less than 0.05. A positive or negative sign indicates a direct or inverse effect, respectively, and the magnitude of the coefficient indicates its relative influence on the response.

2 $${\mathrm{R1}}\,=\,{\mathrm{568}}.{\text{62 }}--{\text{ }}({\mathrm{328}}.{\mathrm{36}} \times {\mathrm{C}}){\text{ }}--{\text{ }}({\mathrm{328}}.{\mathrm{36}} \times {\mathrm{V}}){\text{ }}+{\text{ }}({\mathrm{88}} \times {\mathrm{C}} \times {\mathrm{V}}){\text{ }}+{\text{ }}({\mathrm{48}}.{\mathrm{89}} \times {{\mathrm{C}}^{\mathrm{2}}}){\text{ }}+{\text{ }}({\mathrm{48}}.{\mathrm{89}} \times {{\mathrm{V}}^{\mathrm{2}}})$$

Where R1 = percent of erosion, defined by Eq. 1, C= concentration class (-), and V is the spray rate in L/m².

**Fig. 14:**
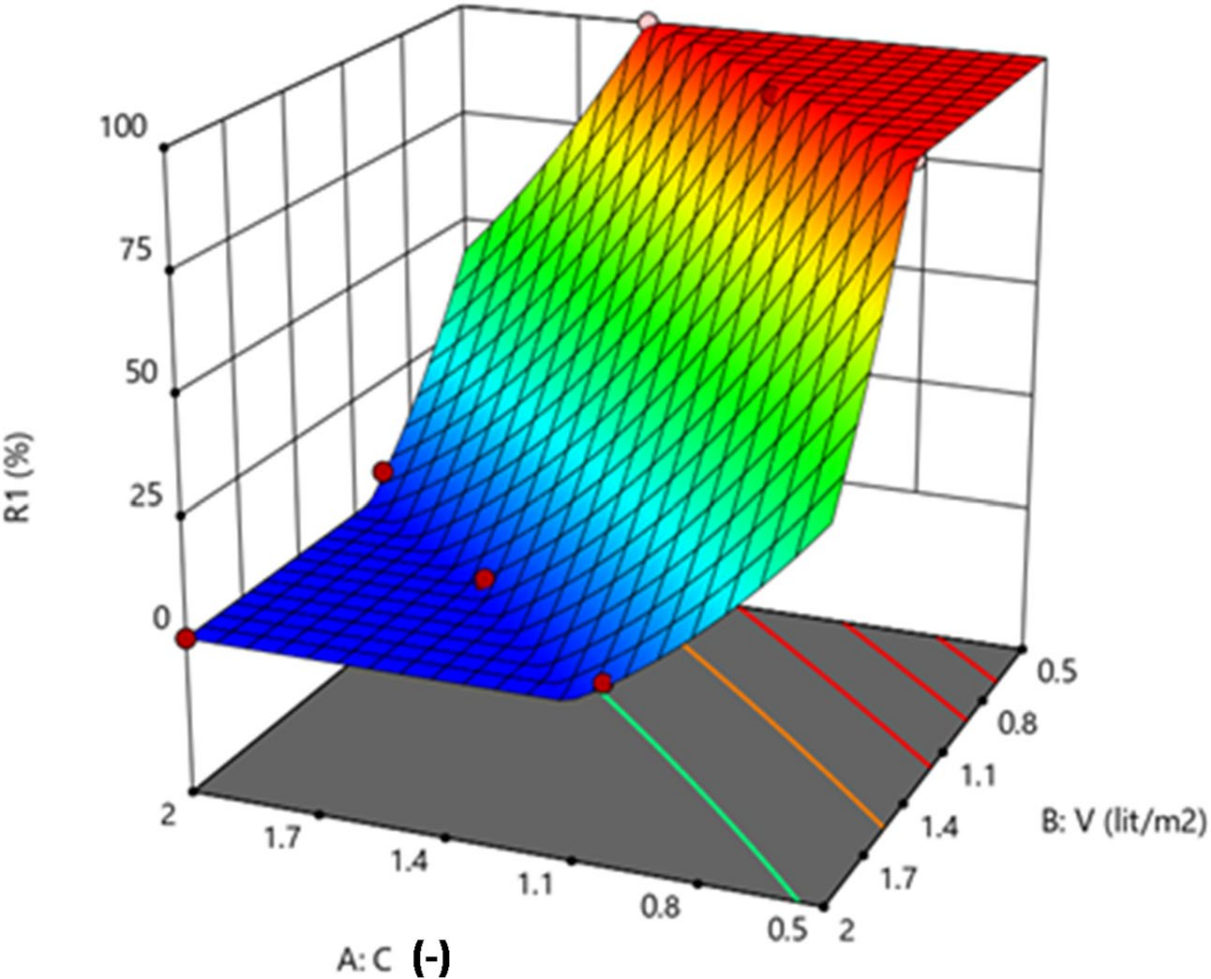
3D surface plot of erosion as a function of concentration class (C) and spray rate (V).

Experimental results presented in Table 8 (Experiment No. 2) indicate that a low spray rate leads to complete erosion of the treated sand due to a reduced penetration depth of the solution. Hence, a thin crust is formed, resulting in rapid cracking and complete sample failure. The 3D surface plot in Fig. 14 shows the combined effect of solution concentration and spray rate on erosion. For optimization purposes, the constraints were set to minimize erosion (target = 0), minimize concentration for economic reasons, and keep the spray rate within its tested range. The software suggested 48 optimal combinations, and the selected combination has a concentration class of 1.39 and a spray rate of 1.65 L/m². This means that the precise quantities of each component required to stabilize 1 square meter of soil are 13.76 g urea, 21.78 g calcium chloride, and 1.43 g zinc Schiff base complex.

### Ancillary tests results

#### Durability test results on stabilized soil

**Fig. 15:**
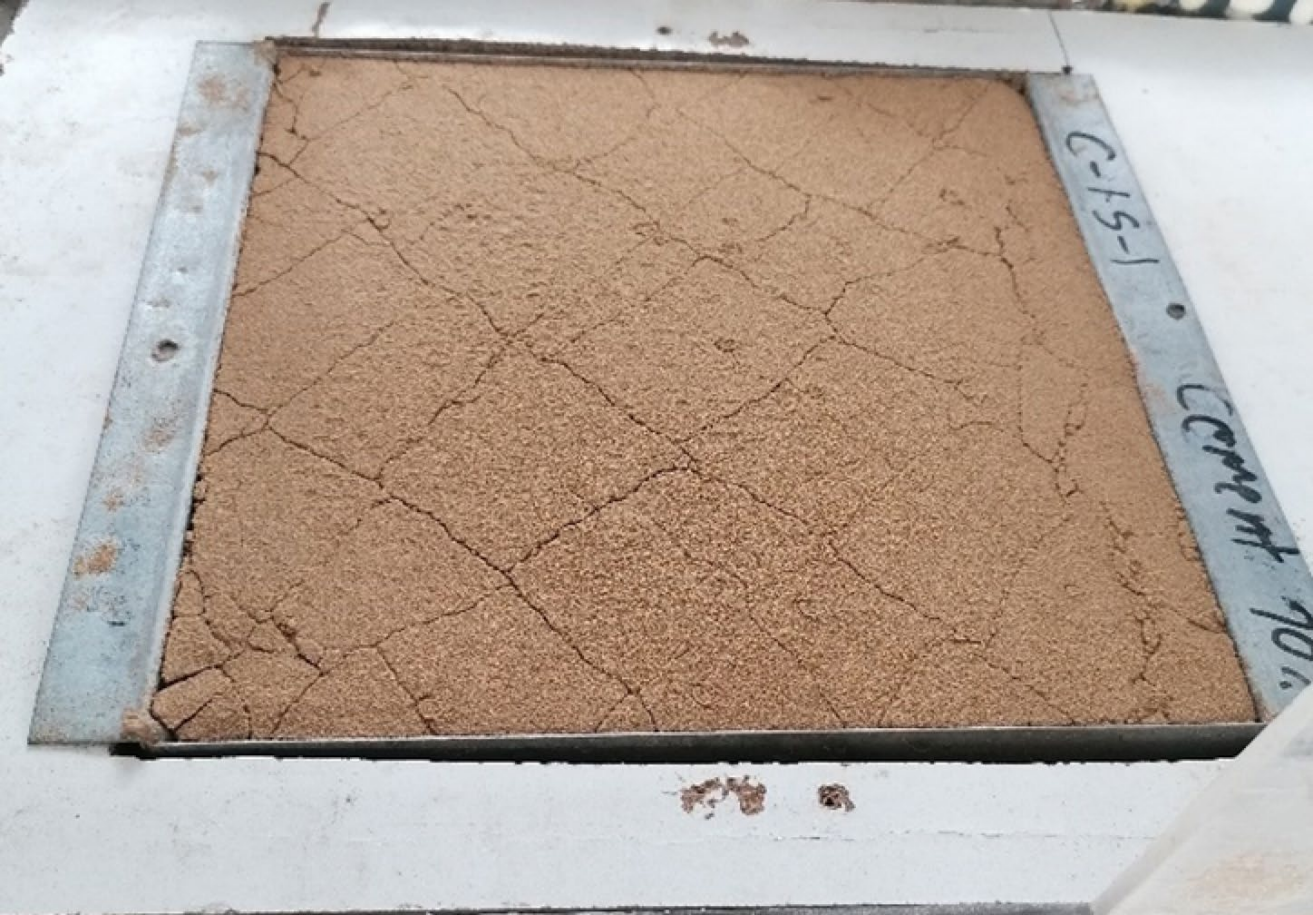
Cracks on the surface of the stabilized soil after the sixth cycle.

**Fig. 16:**
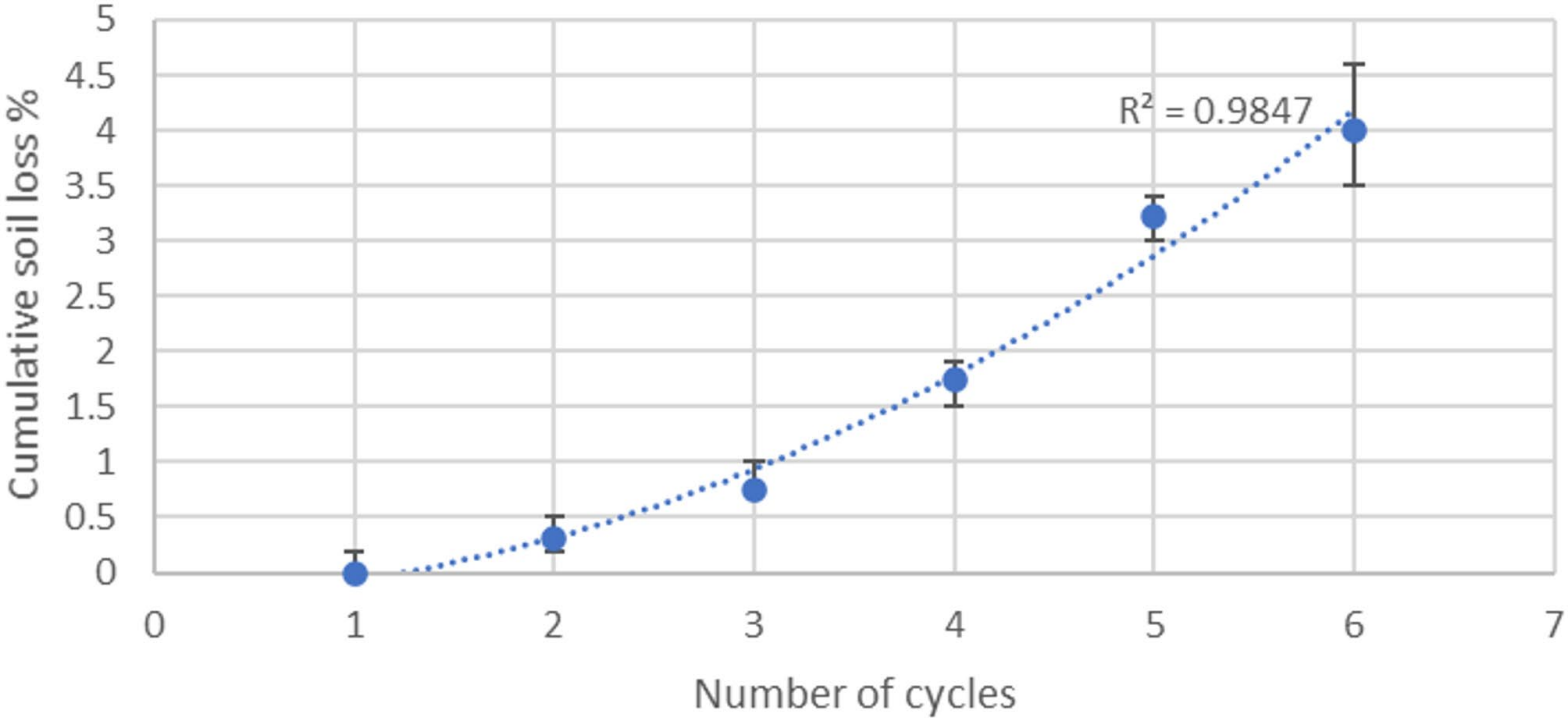
Graph of soil erosion due to wet-dry cycles.

Durability was assessed in wind tunnel tests on soil samples, treated with the selected catalyst solution at the optimum concentration and spray volume, and subjected to numerous wetting-drying cycles. Figure 15 shows the condition of the sample after six cycles, indicating its suitable durability. Figure 16 shows the soil loss of the stabilized soil over consecutive cycles. Maximum erosion of about 4% occurred after the sixth cycle. After the sixth cycle, numerous cracks appeared on the sample, which led to a severe increase in erosion in subsequent cycles.

The durability of the stabilizing agent was also assessed one year after application with the optimal concentration and spray rate. During this period, the sample was stored in the laboratory without controlled temperature or humidity, meaning it was subject to ambient environmental conditions throughout the year. The result of this test is shown in Table 11. As shown in the table, the sample lost a negligible amount of weight (0.5%) after 1 year, indicating the stabilizing material’s suitable durability.

The effect of heat was also investigated by exposing samples stabilized with the optimal concentration and spray rate to 50 °C. The results were compared with samples dried at an outdoor winter temperature of 10 °C. After wind tunnel testing, the results indicate that heat has no significant effect on the wind erosion of soil treated with the zinc Schiff base complex (via calcite precipitation), and the sample remained resistant to erosion (Table 12).

#### Crust characteristics

**Fig. 17:**
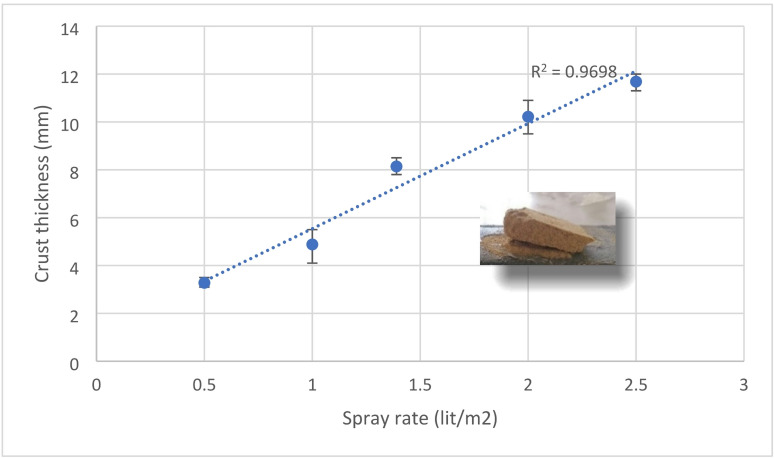
Surface crust thickness versus spray rate.

**Fig. 18:**
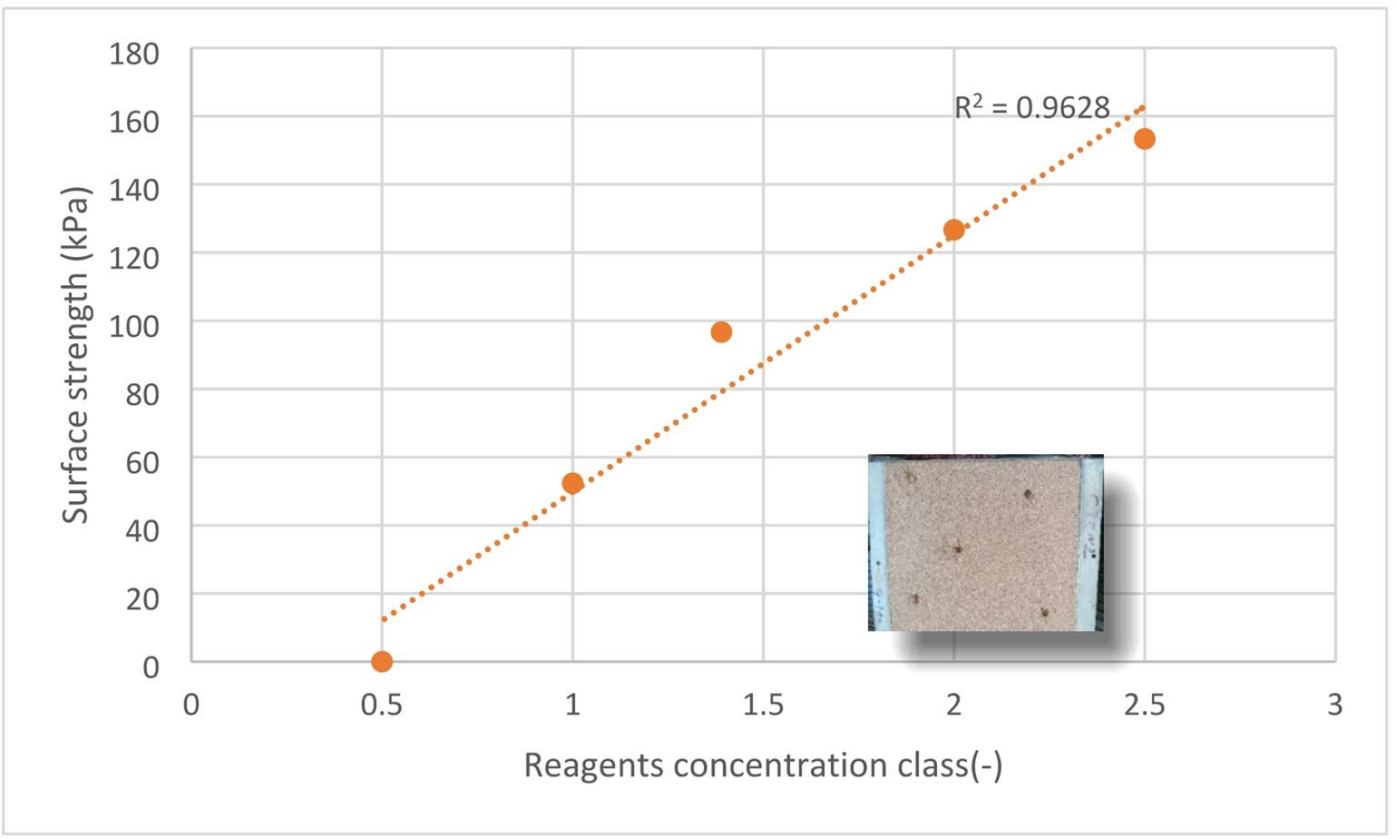
Surface strength for samples stabilized with different solution concentrations (spray rate of 1.65 L/m²).

Crust thickness of the samples treated with a concentration level of 1.39 and different spray rates (0.5, 1, 1.5, 2, and 2.5 L/m²) was measured after the samples were completely dry. The results are shown in Fig. 17. Increasing the spray rate (and thus the penetration depth) resulted in a greater crust thickness, which reduced surface cracking and increased resistance to wind erosion. The minimum crust thickness for the optimum case scenario (i.e. concentration level/code of 1.39) was found to be about 8 mm, as inferred from Fig. 16,

The results of the surface strength test, shown in Fig. 18, indicate an increase in surface strength with increasing solution concentration. The results show that the surface strength can increase to over 150 kPa at higher concentrations and for the spray rate used in this study.

**Table 11:** Erosion after 1 year of stabilization.

Erosion after 10 days (Mean ± SD)	Erosion after 1 year (Mean ± SD)
1.0 ± 0.13%	**1.5** ± **0.1%**

**Table 12:** Effect of heat on wind erosion.

Erosion after drying at 10 °C (Mean ± SD)	Erosion after drying at 50 °C (Mean ± SD)
1.0 ± 0.18%	**1.5** ± **0.16%**

Values are reported as mean ± standard deviation based on three independent replicates.

A threshold detachment velocity of approximately 5 m/s is determined for this soil in its untreated condition^16,61^. For the treated specimens in this investigation, the wind tunnel tests were carried out at the maximum wind speed of 20 m/s. Recent studies have shown surface strength correlates with the threshold detachment velocity^62^. Since the surface strength of the soil was measured at different concentration of solution, an estimate of the threshold velocity may be obtained from the following empirical equation:

$$\:{V}_{t}=C\sqrt{\frac{{q}_{u}}{\rho\:}}$$

**Fig. 19:**
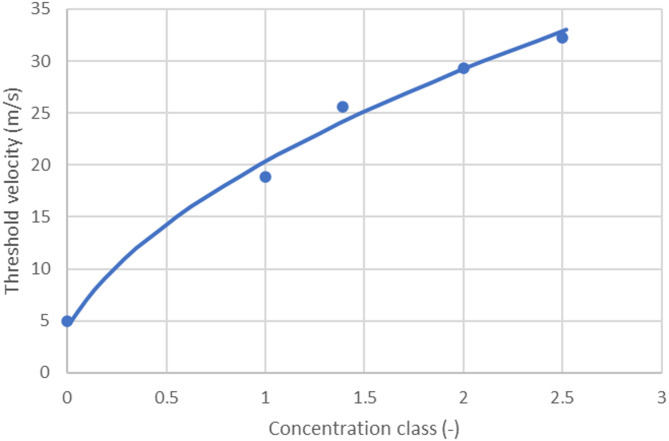
Threshold velocity with different solution concentrations (spray rate of 1.65 L/m²).

Where, q_u_ is the surface shear strength (Pa), and $\:\rho\:$ is the soil density, and C a coefficient ranging within 2 to 5. The type of behavior expressed by this equation confirms well with the previous findings^61^ tested the same soil from the same locality. Using an average value for the parameter C equal to 3.5, the estimated threshold velocity for the tested soil at different concentrations are presented in Fig. 19.

The calcium carbonate content of the crust samples was determined to be varied between 6.1 and 10.9%, for the concentration levels varied between 1.39 and 2.5.

#### Scanning electron microscope (SEM) analysis results

**Fig. 20:**
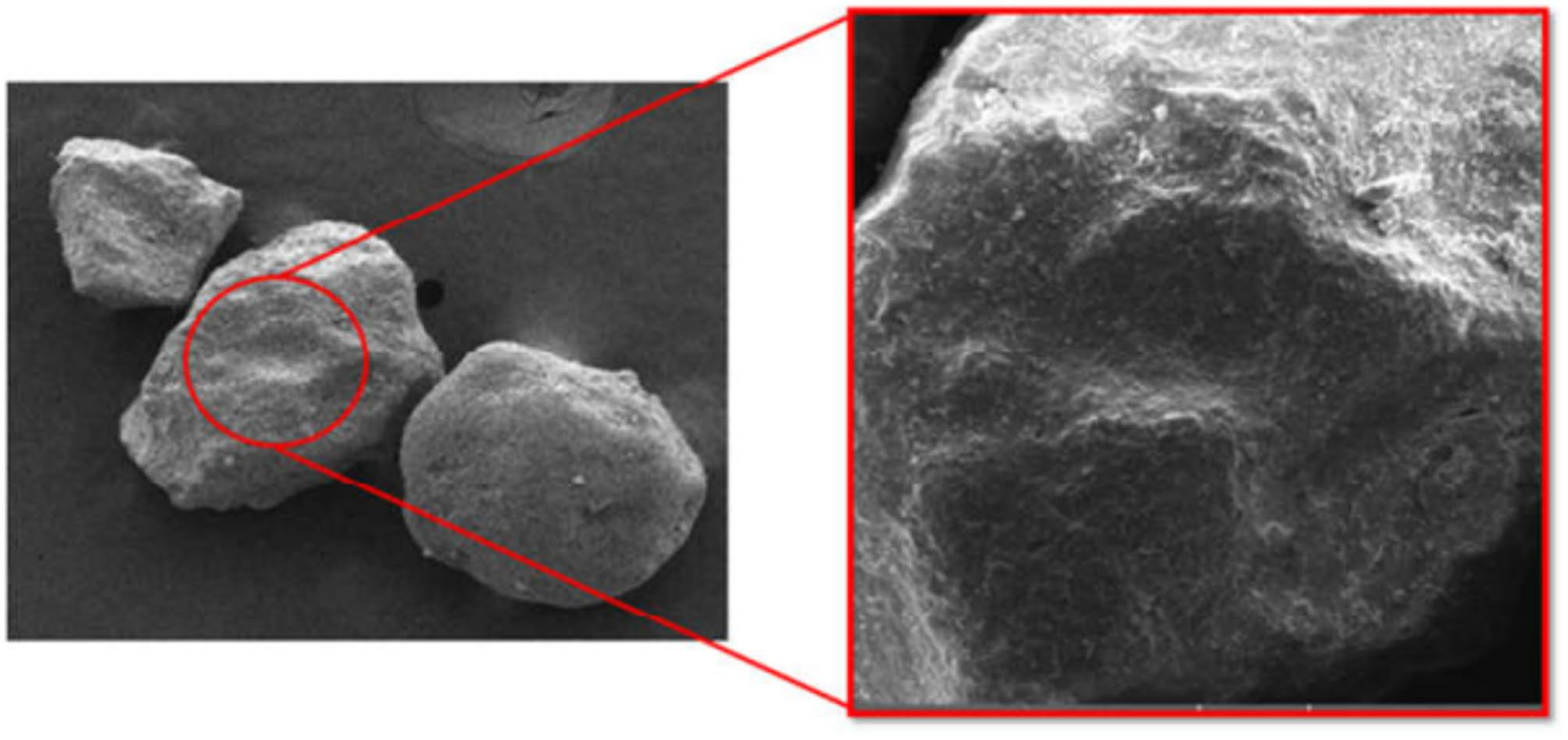
Surface of untreated soil grains.

**Fig. 21:**
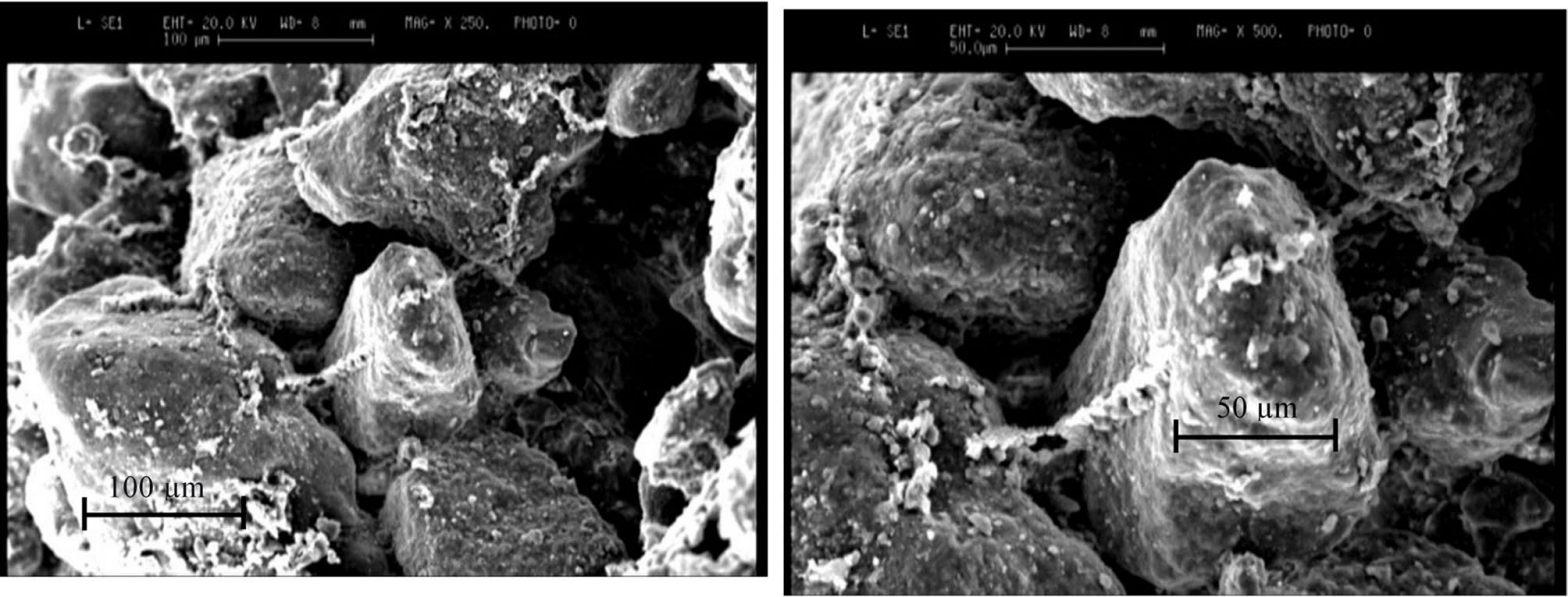
SEM image of stabilized soil sample (a) 250 X magnification, (b) 500 X magnification.

**Fig. 22:**
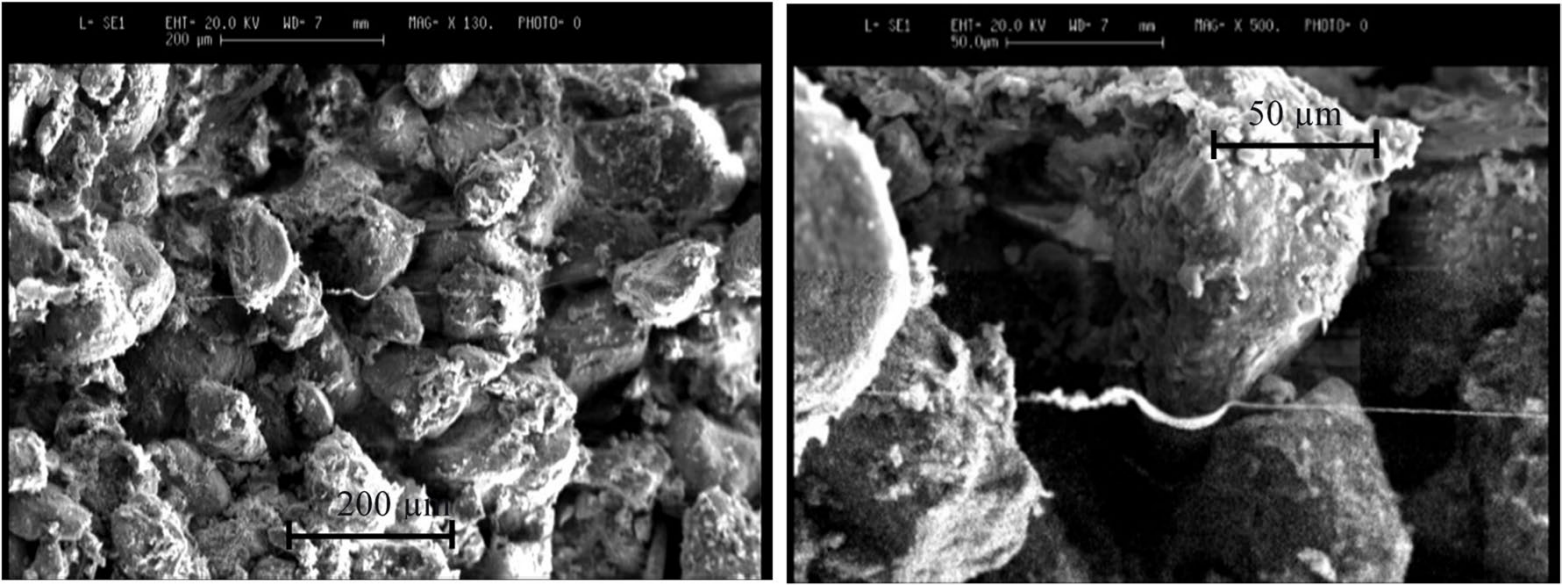
SEM image showing bonding between particles. (**a**) 130 X magnification, (**b**) 500 X magnification.

**Fig. 23:**
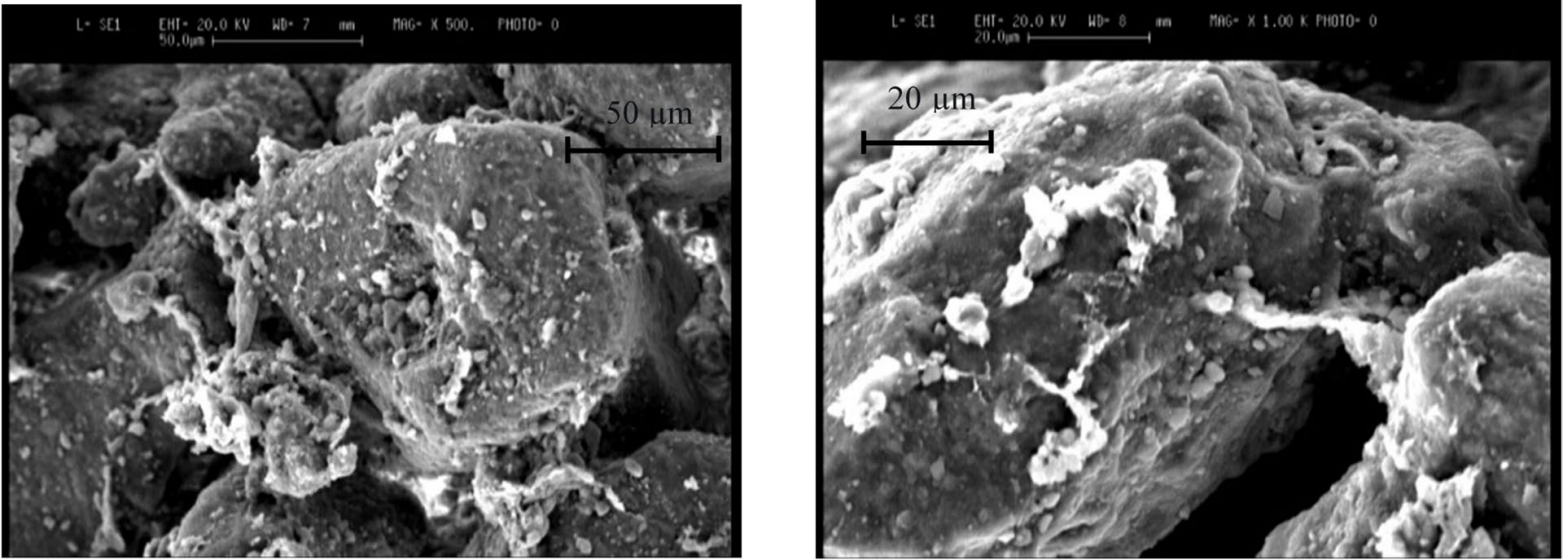
SEM image showing surface coating of grains. (**a**) 500X magnification, (**b**) 1000X magnification.

As discussed earlier in the Sect.  3.1.5, the results of XRD and FTIR analysis confirm production of CaCO_3_ precipitates in the form of calcite crystals. In this section morphology of the precipitates in the curst has been examined. To investigate the mechanism involved in crust formation, SEM analyses were performed and presented in Figs. 20, 21, 22 and 23. As revealed in SEM images at higher magnification, the results indicate the formation of bonds between soil grains (Fig. 21b, and Fig. 22b), and the coating of some grains by calcite crystals under the optimum combination (Fig. 23b). These bonds and coatings increase the cohesion between soil particles and bind particles together. The resulting aggregation increases the weight of soil aggregates, thereby preventing wind erosion.

## Wind erosion mitigation: synthesized biometric urease enzyme versus crude urease enzyme

Table 13 compares the results of this study against some of the previous studies focusing on dust mitigation using EICP (mostly using crude enzyme). As can be seen, from the economical point of view, the presented synthesized enzyme can compete well with 0.32 USD/m^2^ reported by Zhang et al. ^63^. Furthermore, although the amount of penetration resistance achieved by EICP depends on the soil type and reagents’ amount, the penetration resistance achieved in this study stays in the range close to other studies presented in the literature such as Wang et al. ^64^ and Alaufi et al. ^65^. However, there is a discrepancy in the reported values of CaCO_3_ content in the literature associated with similar penetration resistance, which can root in heterogeneity of calcium carbonate precipitation within crust and also the presence of both active and inactive bonds (i.e., calcium carbonate bridges) formed among grains. Therefore, it is not only the total amount of precipitated calcium carbonate crystals but also their effective contribution to dust mitigation and mechanical strength that matters. These issues have also been discussed in previous studies such as Terzis and Laloui^24^ and Soga and Al Qabany^66^. To elucidate the relationship between the precipitation pattern and mechanical strength, the use of imaging techniques such as micro-CT imaging studies deems necessary, which is recommended for future studies. The synthetically biomimetic enzyme provides promising results in increasing wind erosion, while alleviating the need for biological sources. Preliminary cost estimation shows it can reduce the material cost, given low reagents concentrations it requires as well as its reasonable precipitates’ mass to catalyst mass ratio.

**Table 13:** Comparison between performance of EICP through urease and biomimetic (Schiff-base complex) urease enzyme in dust mitigation and soil stabilization.

Soil origin/ type	Urea (M)	CaCl_2_ (M)	Enzyme (conc., g/L)	App. rate (L/m^2^) (cycles)	Detach. velocity (m/s)	Penetration Strength (kPa)	Cost (USD/m^2^)	Calcium carbonate content (%)	Remark	Ref.
Dejgah soil (SP-SM)	0.14	0.09	Schiff-base urease enzyme (1)	1.65 (1)	> 25	96	0.21	6.1	For spray volume of 1.5–2.5 L/m^2^ crust thick.: 8–11.68 mm	This study
Dejgah soil (SP-SM)	0.25	0.16	Schiff-base urease enzyme (1.56)	1.65 (1)	> 32	153	0.38	10.9	-	This study
Yellow river silt, China	1.25	1.25	Crude Soybean urease (N.R.)	4 (1)	14–17	-	0.32	N.R.*	Comp. strength > 725 kPa, crust thick. 14.97 mm	Zhang et al. ^63^
Fallow agricultural soil, Arizona (SM-SC)	1-1.5	0.67–1.2	Crude jack bean urease enzyme (N.R.)	0.8–2.4 (1–2)	11–16	85–581	-	5.9–8.26	Activity of 1000 U/L	Alaufi et al. ^65^
Dust soil from Jiangsu China (Fine silty soil)	0.25	0.25	Crude Soybean urease (100)	2.4 (1)	27	100–200	-	0.75–1.3	Activity of 7000 U/L	Wang et al. ^64^
Arizona Silty sand, medium grained silica sand, mine tailing	1–2	0.375-2	Jack bean urease enzyme (0.5)	4**	25	-	-	-	-	Knorr^38^
Tengger Deseret sand	0.75	0.75 (calcium acetate)	Crude Soybean urease (-)	2.6 (6)	29.1	-	-	-	Activity of 4000 U/L, the cost of the method estimated to be the same as the grass square method	Miao et al. ^67^

*Not reported for the biocrust samples.

** This is for enzyme; a different rate (i.e., 24 L/M^2^) was employed for cementation solution.

## Conclusion

### Summary and concluding remarks

This study successfully developed and validated a novel soil stabilization method using synthetic, enzyme-mimetic Schiff base complexes to catalyze calcite precipitation for wind erosion control. The investigation led to the following key conclusions:

- **Catalyst and precipitate characterization**: Complex bonds and chemical structure were characterized through FTIR, and 1HNMR. The precipitates were confirmed to be calcite through FTIR and XRD spectroscopy.
**- Catalyst Selection and Efficiency**: Between the two synthesized catalysts, the Zinc Schiff base complex was identified as the more effective and economically viable catalyst for urea hydrolysis and subsequent calcite precipitation, outperforming the Copper complex in yield and cost-effectiveness.
**- Optimal Treatment Formula**: A definitive optimal treatment recipe was established empirically and statistically. The most proposed stabilizing solution consists of 0.12 g urea and 0.19 g calcium chloride per 10 ml of water, catalyzed by 0.0125 g of the Zinc complex. Scaled up, this equates to 8 g/L urea, 13 g/L CaCl_₂_, and 1 g/L Zn-complex.
**- Optimal Application**: Based on the Response Surface Methodology (RSM) analysis and the results from the wind tunnel tests, it was determined that the aforementioned solution must be applied at a rate of 1.65 L/m² to achieve maximum effectiveness. This specific spray volume ensures sufficient penetration and crust formation without inducing desiccation cracks that compromise stability, as confirmed by the experiments.
**- Effective Erosion Control**: Wind tunnel tests demonstrated that the optimized treatment could reduce soil erosion in a highly erodible sand to nearly zero (~ 1%), providing exceptional resistance to wind forces.
**- Durability and Performance**: The formed calcite crust exhibited significant durability against environmental challenges. It maintained high performance through multiple wet-dry cycles, under elevated temperatures (50 °C), and after one year of aging in ambient laboratory conditions, with only a minimal reduction in effectiveness.
**- Mechanism of Stabilization**: SEM analysis confirmed the mechanism of stabilization, showing that the precipitated calcite effectively coats soil grains and forms bridges between them, increasing cohesion and aggregate weight, which is the primary reason for the enhanced resistance to erosion.

In conclusion, this research presents a significant advancement in the field of bio-inspired ground improvement. The use of synthetic zinc-Schiff base complexes as urease mimics proves to be a robust and durable alternative to the biological EICP and MICP methods. The findings provide a practical and reliable solution for mitigating dust emissions from susceptible soils, with immediate potential for application in arid and semi-arid regions impacted by wind erosion. Future work could focus on field-scale pilot studies and further investigation into the long-term environmental impact of the catalysts.

### Limitations and suggestions for future works

While this study demonstrates the significant potential of synthetic Schiff base complexes for soil stabilization, several limitations should be acknowledged to contextualize the findings and guide future research:

- **Laboratory-Scale Conditions**: All experiments were conducted under controlled laboratory conditions. The performance and durability of the treatment may vary significantly in the field, where factors such as fluctuating humidity, extreme temperature cycles, UV radiation, and variable wind patterns and intensities are present.

Also, the solution was applied manually via sprayer to small, prepared samples. Scaling this application method to large, uneven field sites poses significant practical and economic challenges. The feasibility of using agricultural sprayers or irrigation systems for large-scale application needs to be evaluated.

- **Field-scaling implementation**:

Field and pilot studies were beyond the scope of present study and the effects of challenges such as uneven topography and infiltration variability have to be considered in future studies. Although the field implementation of urease based EICP has received limited attention recently by recent studies such as Li et al. and Wang et al.^68,69^, the field implementation of the introduced Schiff base complexes remains underexplored and warrants further studies.

- **Durability**:

The durability evaluation focused on comparing initial performance with retained performance after one year of ambient aging. Intermediate time intervals were not monitored, and future studies should incorporate multiple aging stages to better capture degradation trends over time. Various chemical additives^70^, biopolymers^71^ and methods introduced in recent years to enhance thermal durability of natural urease enzyme^72^ can also be employed to enhance durability of Schiff base complexes. This provides another research direction for future studies.

- **Soil Specificity**: The research was conducted on a single type of soil (a poorly graded silty sand, SP-SM). The efficacy of the treatment on other soil types, particularly those with high clay or silt content, different mineralogy, or higher salinity, remains untested and is an area for future investigation.
- **Economic Analysis**: While the Zinc complex was selected for its lower cost relative to the Copper complex and purified enzymes (as illustrated in Table 13), a detailed life cycle assessment and economic analysis comparing this method to other stabilization techniques (e.g., traditional mulches, biopolymers, or chloride salts) is encouraged in future studies.
- **Investigating higher wind speed**,** and saltation phenomena**:

This study was focused on studying the dust suppression performance of stabilized soil at wind speed velocity of 20 m/s; although a relationship was established based on the results of this study and previous studies on the same soil type relating detachment wind velocity and surface strength of biocemented soils, experimental assessment of the biomimetric urease enzyme at higher wind speeds is essential. Furthermore, the performance of the stabilized soils under sand bombardment (i.e., saltation) has to be studied in future studies.

Future studies could aim to address the limitations mentioned above. Additionally, the following research directions are proposed:

Integrating the proposed catalyst with complementary methods, such as biopolymers, could be explored to enhance treatment durability and address economic concerns.

Investigating the effect of synthetic Schiff base complexes on key geotechnical properties—including shear strength, compressibility, and permeability—would be valuable. This would broaden the method’s application to other engineering problems, such as improving bearing capacity and slope stability.

## Supplementary Information

Below is the link to the electronic supplementary material.

Supplementary Material 1

## Data Availability

Data would be available upon request from the corresponding author.

## References

[1] Deylaghian, S., Nikooee, E., Habibagahi, G. & Nagel, T. Inulin biopolymer as a novel material for sustainable soil stabilization. *Sci. Rep.* **14** (1), 31078 (2024). 39730738 10.1038/s41598-024-82289-8 PMC11680679

[2] Mohebbi, M. M., Habibagahi, G., Niazi, A. & Ghahramani, A. A laboratory investigation of suppression of dust from wind erosion using biocementation with Bacillus amyloliquefaciens. *Scientia Iranica*. **26** (5), 2665–2677 (2019).

[3] Morales, C. Saharan dust: mobilization, transport, deposition. SCOPE Series (France) eng no. 14. (1979).

[4] Sterk, G. Causes, consequences and control of wind erosion in Sahelian Africa: a review. *Land. Degrad. Dev.* **14** (1), 95–108 (2003).

[5] Goudie, A. S. & Middleton, N. J. *Desert dust in the global system* (Springer Science & Business Media, 2006).

[6] Padmakumar, G. P. et al. Characterization of aeolian sands from Indian desert. *Eng. Geol.* **139**, 38–49 (2012).

[7] Devrani, R., Dubey, A. A., Ravi, K. & Sahoo, L. Applications of bio-cementation and bio-polymerization for aeolian erosion control. *J. Arid Environ.* **187**, 104433 (2021).

[8] Dubey, A. A. et al. Experimental investigation to mitigate aeolian erosion via biocementation employed with a novel ureolytic soil isolate. *Aeolian Res.* **52**, 100727 (2021).

[9] Johnson, A. M. The climate of Peru, Bolivia, and Ecuador (Ch. 4). *Climates Cent. South. Am.* **12**, 147–218 (1976).

[10] Shao, Y. *Physics and modelling of wind erosion* Vol. 37 (Springer Science & Business Media, 2008).

[11] Liu, Y. et al. Microbial-induced calcium carbonate precipitation: Influencing factors, nucleation pathways, and application in waste water remediation. *Sci. Total Environ.* **860**, 160439. 10.1016/j.scitotenv.2022.160439 (2023). 36574549 10.1016/j.scitotenv.2022.160439

[12] Armbrust, D. V., Chepil, W. S. & Siddoway, F. H. Effects of ridges on erosion of soil by wind. *Soil Sci. Soc. Am. J.* **28** (4), 557–560 (1964).

[13] Vaezi, A. R., Bahrami, H. A., Sadeghi, S. H. R. & Mahdian, M. H. Modeling relationship between runoff and soil properties in dry-farming lands, NW Iran. *Hydrol. Earth Syst. Sci. Dis.* **7** (2), 2577–2607 (2010).

[14] Afrin, H. A review on different types soil stabilization techniques. *Int. J. Transp. Eng. Technol.* **3** (2), 19–24 (2017).

[15] Bouhicha, M., Aouissi, F. & Kenai, S. Performance of composite soil reinforced with barley straw. *Cem. Concr. Compos.* **27** (5), 617–621 (2005).

[16] Owji, R., Habibagahi, G., Nikooee, E. & Afzali, S. F. Wind erosion control using carboxymethyl cellulose: From sand bombardment performance to microfabric analysis. *Aeolian Res.* **50**, 100696 (2021).

[17] Whiffin, V. S. Microbial CaCO_3_ Precipitation for production of Biocement. PhD Dissertation in Biotechnology, Murdoch University. (2004).

[18] Hemayati, M., Nematollahi, A., Nikooee, E., Habibagahi, G. & Niazi, A. Non-ureolytic microbially induced carbonate precipitation: Investigating a cleaner biogeotechnical engineering pathway for soil mechanical improvement. *J. Eng.* **2024** (1), e12350 (2024).

[19] Price, G. Interview. Melbourne, Australia. (2012).

[20] Zhang, J. et al. Strength and uniformity of EICP-treated sand under multi-factor coupling effects. *Biogeotechnics* **1** (1), 100007. 10.1016/j.bgtech.2023.100007 (2023).

[21] Dejong, J. T. et al. T. Biogeochemical processes and geotechnical applications: progress, opportunities and challenges. In *Bio-and chemo-mechanical processes in geotechnical engineering: géotechnique symposium* in print 2013 (pp. 143–157). (Ice Publishing, 2014).

[22] Sadjadi, M., Nikooee, E. & Habibagahi, G. Biological treatment of swelling soils using microbial calcite precipitation. In *Unsaturated Soils: Research and Applications* (Khalili, N. ed) 917–922. (CRC, London, 2014).

[23] Moravej, S., Habibagahi, G., Nikooee, E. & Niazi, A. Stabilization of dispersive soils by means of biological calcite precipitation. *Geoderma* **315**, 130–137 (2018).

[24] Terzis, D. & Laloui, L. 3-D micro-architecture and mechanical response of soil cemented via microbial-induced calcite precipitation. *Sci. Rep.* **8** (1), 1416 (2018). 29362386 10.1038/s41598-018-19895-w PMC5780488

[25] Saffari, R., Habibagahi, G., Nikooee, E. & Niazi, A. Biological stabilization of a swelling fine-grained soil: The role of microstructural changes in the shear behavior. *IJST* **41** (4), 405–414. 10.1007/s40996-017-0066-z (2017).

[26] Saffari, R., Nikooee, E., Habibagahi, G. & Van Genuchten, M. T. Effects of biological stabilization on the water retention properties of unsaturated soils. *J. Geotech. GeoEnviron. Eng.* **145** (7), 04019028 (2019).

[27] Afzali, S. F., Hemayati, M., Niazi, A. & Nikooee, E. Toward industrialization of microbially induced carbonate precipitation for wind erosion suppression: Novel methodology, challenges, and opportunities. *Iran. J. Sci. Technol. Trans. Civil Eng.* **48** (2), 1143–1149 (2024).

[28] Devrani, R., Vangla, P. & Sharma, S. Exploring the Potential of Native Urease-Producing Bacteria of Hilly Terrain for Soil Strength Enhancement. *Geomicrobiol J.* **41** (8), 818–829 (2024).

[29] Niknam Safari Kouchi, E., Nikooee, E., Habibagahi, G., Niazi, A. & Nagel, T. The swelling characteristics of an unsaturated bio-cemented sand-bentonite mixture: Analyzing the effect of bacterial concentration and suction. *Iran. J. Sci. Technol. Trans. Civ. Eng.* **49** (4), 1–21 (2025).

[30] Al-Thawadi, S. *High strength in-situ biocementation of soil by calcite precipitating locally isolated ureolytic bacteria* (Doctoral dissertation, Murdoch University). (2008).

[31] Wang, Z., Zhang, N., Ding, J., Lu, C. & Jin, Y. Experimental study on wind erosion resistance and strength of sands treated with microbial-induced calcium carbonate precipitation. *Advances in Materials Science and Engineering*, **2018**. (2018).

[32] Dubey, A. A., Dhami, N. K., Ravi, K. & Mukherjee, A. Erosion mitigation with biocementation: a review on applications, challenges, & future perspectives. *Reviews Environ. Sci. Bio/Technology*. **22** (4), 1059–1091 (2023).

[33] Van Wijngaarden, W. K., Vermolen, F. J., Van Meurs, G. A. M. & Vuik, C. A mathematical model and analytical solution for the fixation of bacteria in biogrout. *Transp. Porous Media*. **92** (3), 847–866 (2012).

[34] Wang, Y. et al. State-of-the-art review of soil erosion control by MICP and EICP techniques: Problems, applications, and prospects. *Sci. Total Environ.* **912**, 169016. 10.1016/j.scitotenv.2023.169016 (2024). 38043825 10.1016/j.scitotenv.2023.169016

[35] Van Paassen, L. A. et al. Scale up of BioGrout: a biological ground reinforcement method. In *Proceedings of the 17th International Conference on Soil Mechanics and Geotechnical Engineering (Volumes 1, 2, 3 and 4)* (pp. 2328–2333). (IOS Press, 2009).

[36] Barkouki, T. H. et al. Forward and inverse bio-geochemical modeling of microbially induced calcite precipitation in half-meter column experiments. *Transp. Porous Media*. **90** (1), 23–39 (2011).

[37] Taharia, M. et al. Microbial induced carbonate precipitation for remediation of heavy metals, ions and radioactive elements: A comprehensive exploration of prospective applications in water and soil treatment. *Ecotoxicol. Environ. Saf.* **271**, 115990. 10.1016/j.ecoenv.2024.115990 (2024). 38262090 10.1016/j.ecoenv.2024.115990

[38] Knorr, B. *Enzyme-induced carbonate precipitation for the mitigation of fugitive dust* (Arizona State University, 2014).

[39] Almajed, A. et al. Enzyme-Induced Carbonate Precipitation (EICP)-Based methods for ecofriendly stabilization of different types of natural sands. *J. Clean. Prod.* **274**, 122627 (2020).

[40] Mobarezi, M., Nikooee, E., Owji, R. & Habibagahi, G. Enzyme-induced carbonate precipitation as a novel remedy for expansive soils: assessing microfabric and swelling characteristics. *Geotech. Geol. Eng.* **42** (7), 6457–6475 (2024).

[41] Dagliya, M. & Satyam, N. Optimization of urease amount for calcite precipitation in indian desert sand using biologically inspired method. *Indian Geotech. J.* **55** (3), 1–12 (2024).

[42] Almajed, A., Lemboye, K., Arab, M. G. & Alnuaim, A. Mitigating wind erosion of sand using biopolymer-assisted EICP technique. *Soils Found.* **60** (2), 356–371 (2020).

[43] Dagliya, M., Satyam, N. & Garg, A. Biopolymer based stabilization of Indian desert soil against wind-induced erosion. *Acta Geophys.* **71** (1), 503–516 (2023).

[44] Nassr, L. A. M. E. & Abu-Dief, A. M. Kinetic Screening for the Acid‐Catalyzed Hydrolysis of Some Hydrophobic Fe (II) Schiff Base Amino Acid Chelates and Reactivity Trends in the Presence of Alkali Halide and Surfactant. *Int. J. Chem. Kinet.* **47** (8), 501–508 (2015).

[45] Mohamad, A. D. M., Abualreish, M. J. A. & Adam, M. S. S. Kinetics of the base hydrolysis of iron (II) complexes with pyridyl–quinolyl Schiff base ligands in aqueous and aqueous/methanol binary mixtures. *J. Iran. Chem. Soc.* **12** (9), 1521–1528 (2015).

[46] Islam, N., Rahman, H. & Lutfor Rahman, M. Kinetics and mechanism of hydrolysis of urea and N, N′-Diacetyl of urea in presence of Cobalt (II), Copper(II), Zink(II)-Schiff base complexes. *Int. J. Adv. Res.* **6** (1), 521–529 (2018).

[47] Blakeley, R. L., Treston, A., Andrews, R. K. & Zerner, B. Nickel (II)-promoted ethanolysis and hydrolysis of N-(2-pyridylmethyl) urea. A model for urease. *J. Am. Chem. Soc.* **104** (2), 612–614 (1982).

[48] Banu, S. Catalytic activities of Zn(II), Co(II), Ni(II), Cu(II), Ru(II), Pd(II) and Mn(II) towards the hydrolysis of urea, M.Sc Thesis, Department of Chemistry, University of Rajshai, Bangladesh. (1988).

[49] Chakraborty, H., Paul, N. & Rahman, M. L. Catalytic activities of Schiff base aquocomplexes of copper(II) towards hydrolysis of amino acid esters. *Transition Met. Chem.* **19**, 524–526. 10.1007/BF00136366 (1994).

[50] Salma, U., Alam, M. Z. & Ahmad, S. A Comprehensive Review on Recent Advances of Remarkable Scaffold Triazole based Schiff Base: Synthesis and Photoresponsive Chemosensors for Al^3+^ Ion Detection. *J. Fluoresc*. 10.1007/s10895-025-04202-4 (2025). 40117055 10.1007/s10895-025-04202-4

[51] Faghihinia, M. & Afzali, S. F. Effects of wind erosion on soil organic carbon dynamics and other soil properties: Dejgah catchment, Farashband County, Shiraz Province, Iran. *Afr. J. Agric. Res.* **8** (34), 4452–4459 (2013).

[52] Ayeldeen, M., Negm, A., El Sawwaf, M. & Gädda, T. Laboratory study of using biopolymer to reduce wind erosion. *Int. J. Geotech. Eng.* **12** (3), 228–240 (2018).

[53] Tourtiz, A., Mokhberi, M. & Nasehi, S. A. Mitigation of wind erosion using alkali-activated recycled glass powder: an experimental and microstructural study. *Eng. Rep.* e70552. 10.1002/eng2.70552 (2025). 7.

[54] https://www.irimo.ir/eng/index.php

[55] https://www.theguardian.com/world/2025/jul/22/iran-limit-water-temperature-50c-and-reservoirs-depleted-extreme-heat-drought

[56] https://www.weatherandradar.co.uk/weather-news/global-records-fall-extreme-heat-around-the-world--12d939f5-686f-4dd7-abb6-27c9d0e8a65b

[57] Butera, V. et al. How the metal ion affects the ^1^H NMR chemical shift values of Schiff base metal complexes: rationalization by DFT calculations. *J. Phys. Chem. A*. **127** (44), 9283–9290 (2023). 37906682 10.1021/acs.jpca.3c05653 PMC10641838

[58] Kaminskaia, N. V. & Kostić, N. M. Kinetics and mechanism of urea hydrolysis catalyzed by palladium (II) complexes. *Inorg. Chem.* **36** (25), 5917–5926 (1997). 11670215 10.1021/ic961500p

[59] Kaminskaia, N. V. & Kostić, N. M. Alcoholysis of urea catalyzed by palladium (II). *Complexes Inorg. chemistry*. **37** (17), 4302–4312 (1998). 10.1021/ic980065r 11670566

[60] Ahenkorah, I., Rahman, M. M., Karim, M. R. & Teasdale, P. R. Optimization of enzyme induced carbonate precipitation (EICP) as a ground improvement technique. In *Geo-Congress, February, 2020* (552–561). American Society of Civil Engineers, (Reston, VA, 2020).

[61] Hemayati, M., Nikooee, E., Habibagahi, G., Niazi, A. & Afzali, S. F. New non-ureolytic heterotrophic microbial induced carbonate precipitation for suppression of sand dune wind erosion. *Sci. Rep.* **13** (1), 5845 (2023). 37037897 10.1038/s41598-023-33070-w PMC10086056

[62] Scott, B. et al. Abiotic crust formation in fallow agricultural desert soils through carbonate cementation reduces fugitive dust. *Cambridge Prisms: Drylands*, **2**, e3. (2025)

[63] Zhang, J. et al. Bio-Gel Formation Through Enzyme-Induced Carbonate Precipitation for Dust Control in Yellow River Silt. *Gels* **11** (6), 452. 10.3390/gels11060452 (2025). 40558751 10.3390/gels11060452 PMC12192366

[64] Wang, H. et al. Erosion resistance of treated dust soils based on the combined enzymatically induced carbonate precipitation and polyacrylic acid. *Biogeotechnics* **1** (4), 10005065 (2023).

[65] Alaufi, S., Emmanuel, S. & Kavazanjian, E. Optimization of EICP treatment parameters for dust mitigation. *ICBBG conference*, 2025. 10.53243/ICBBG2025-93 (2025).

[66] Al Qabany, A., Soga, K. & Santamarina, C. Factors affecting efficiency of microbially induced calcite precipitation. *J. Geotech. GeoEnviron. Eng.* **138** (8), 992–1001 (2012).

[67] Miao, L., Wu, L. & Sun, X. Enzyme-catalysed mineralisation experiment study to solidify desert sands. *Sci. Rep.* **10** (1), 10611 (2020). 32606324 10.1038/s41598-020-67566-6 PMC7327045

[68] Li, C., Li, S., Xing, J., Gao, Y. & Yao, D. On-site test of fixed mobile sand dunes using combined technology of sand plants and EICP in the Ulanbuh Desert, China. *Acta Geotech.* **20** (4), 1921–1934 (2025).

[69] Wang, H. et al. Sand and dust storms control for sustainable anti-desertification: large-scale EICP-PVAc treatment field demonstration and insights. *Acta Geotech.* **20** (5), 2201–2219 (2025).

[70] Wang, H. et al. The use of N-(N-butyl)-thiophosphoric triamide to improve the efficiency of enzyme induced carbonate precipitation at high temperature. *Acta Geotech.* **18** (9), 5063–5081 (2023).

[71] Wang, H., Sun, X., Miao, L., Cao, Z. & Guo, X. Garlic extract addition for soil improvement at various temperatures using enzyme-induced carbonate precipitation (EICP) method. *J. Rock Mech. Geotech. Eng.* **15** (12), 3230–3243 (2023).

[72] Hemayati, M. et al. A pore-scale study of fracture sealing through enzymatically-induced carbonate precipitation (EICP) method demonstrates its potential for CO2 storage management. *Sci. Rep.* **14** (1), 17832 (2024). 39090349 10.1038/s41598-024-68720-0 PMC11294598

