# Supplementary material for: A synthetic urease-mimetic catalyst for wind erosion control via carbonate precipitation
Source: Sci Rep. 2026 May 14;16:22021. doi: 10.1038/s41598-026-52936-3 (PMC13365427; doi:10.1038/s41598-026-52936-3)
Supplement: Supplementary file 1 — Supplementary Material 1 [file 41598_2026_52936_MOESM1_ESM.docx]

The diffraction experiments and the structure refinement are summarized in Table S1. The selected bond lengths and angles are given in Table S2.

One molecule of the Cu and two water molecules constitute the asymmetric unit of the reported structure (Fig. S1). The O1W is coordinated to the Cu canter. In turn, O2W is positioned on the 2-fold axis, and is involved only in H-bonds to O3 of the glycine moiety and O1W.

The presence of carboxylic group in the glycine fragment results in its participation in two coordination bonds, one intramolecular formed by O2, and the intermolecular Cu1-O3. The series of these intermolecular bonds results in the formation of the infinite chains of the complex molecules along the Y axis, with O3 bridging the adjacent metal centers (Fig. S2).

In the reported structure, the Cu ion has a square-pyramid CuN1O4 coordination sphere. The base of the pyramid is formed by the three-dentate glysal ligand and O1W water molecule. The Cu1-O1 and Cu1-O2 bonds formed by the phenolic O1 and carboxylate O2 are similar in length to that formed by the glycine N1, the respective distances being 1.9173(12), 1.9589(12) and 1.9333(15) Å. The Cu1-O1W bond of 2.0168(13) Å is slightly longer, what reflects the weaker electrostatic component of this interaction and the rigidity of the three-dentate coordination of glysal. The Cu1 coordination is completed by O3[-x+1/2,y-1/2,-z+1/2] atom of the glycine moiety of neighboring complex molecule, with the bond length Cu1-O3[-x+1/2,y-1/2,-z+1/2] of 2.3283(14) Å, the longest in the coordination sphere. Within the base of the square-pyramidal coordination sphere, the angles between bonds formed by the donor atoms in cis positions range from N1-Cu1-O2 83.76(6)° to O1-Cu1-N1 93.88(6)°, and those related to the trans positions are O1-Cu1-O2 165.91(6)° and N1-Cu1-O1W 165.80(6)°. The displacement of the Cu1 ion from the pyramid base is 0.225 Å. The bond angles between the axial bond Cu1-O3[-x+1/2,y-1/2,-z+1/2] and the equatorial bonds are 86.77(5) to 105.88(6)°.

The valence geometry of the glysala ligand is typical for such compounds. The phenolic O1-C1 bond is 1.330(2) Å, the carboxylate O2 and O3 form bonds to C9 with the respective distances of 1.268(2) and 1.242(2) Å. The C7-N1 and N1-C8 bonds are 1.277(2) and 1.461(2) Å, reflecting position of the single and double bond. Within the glysala ligand, two planar fragments can be defined - the phenolate-C7-N1 fragment and C8-C9-O2-O3 carboxylate. The dihedral angle between the best planes is 20.81°. In the conformation found in the structure, the torsion angles C7-N1-C8-C9 and N1-C8-C9-O2 are 154.9(2) and 14.5(2)°.

In the found conformation, the chelate ring Cu1-O2-C9-C8-N1 formed by the glycine moiety is envelope on N1. The ring Cu1-O1-C1-C6-C7-N1 is flat with the torsion angles ranging from 2 to 8° (absolute values).

The crystal packing reveals the network of H-bonds involving the water molecules and the carboxylate O2, O3 atoms. Water O1W is a donor in the H-bonds to O1[-x+1,y,-z+1/2] and O2W[x+1/2,y+1/2,z], with the respective O…O distances being 2.6302(18) and 2.952(2) Å. Water molecule O2W is a donor in the O2W…O3 H-bond, with the O…O distance of 2.9365(17) Å. The C2-H2A…O3[x+1/2,y-1/2,z] and C8-H8B…O2[-x+1/2,y-1/2,-z+1/2] interactions are also detected, with the C…O distances of 3.462(2) and 3.200(2) Å. The π- π interactions are found, involving the phenyl C1—C6 moieties, with the distance between the ring gravity centers Cg…Cg[1-x,-y,1-z] being 3.6799(12) Å.


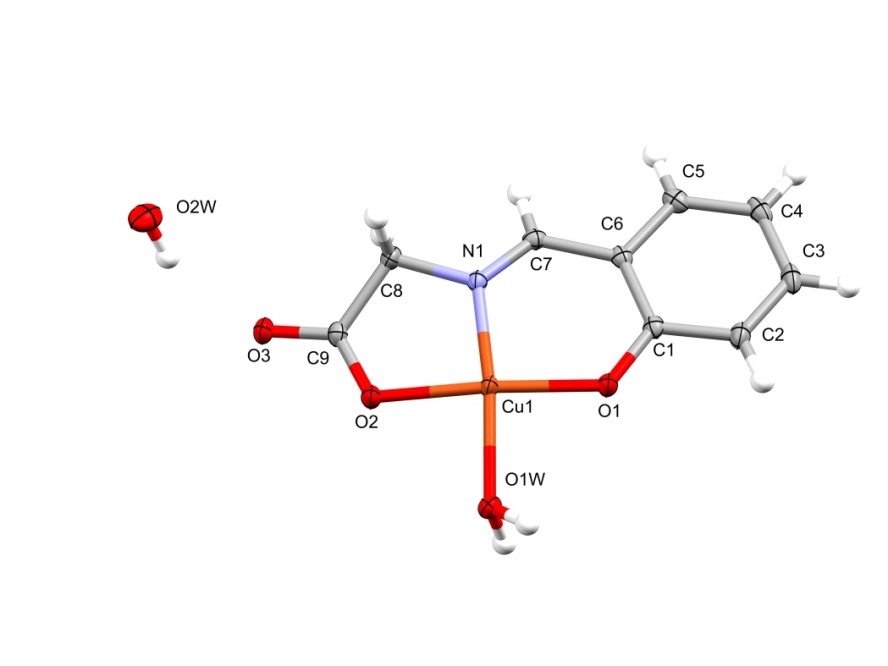


**Figure S1.** Asymmetric unit of the Cu structure.


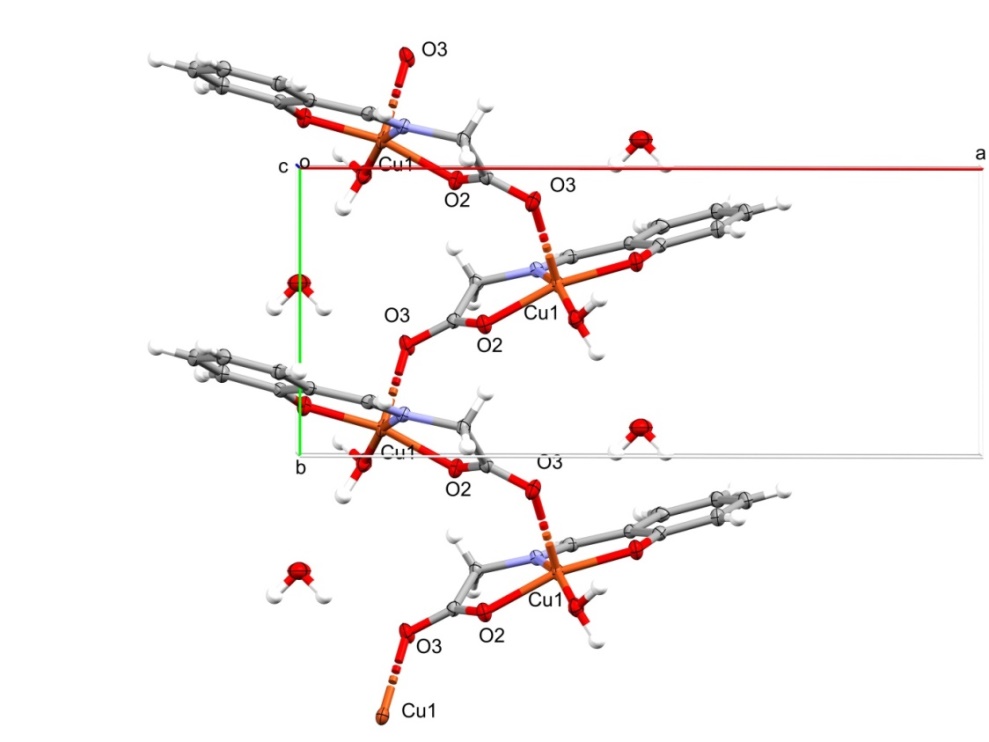


**Figure S2**. Infinite chain along Y direction, formed by the Cu molecules. The glycine O3 atoms bridge the adjacent complex molecules.

**Table S1**. Crystal data and structure refinement for Cu complex.

| Identification code | cuglysala_a |
| --- | --- |
| Empirical formula | C_9_ H_9_ Cu N O_5_ |
| Formula weight | 274.71 |
| Temperature; K | 293(2) |
| Wavelength ; Å | 1.54184 |
| Crystal system | Monoclinic |
| Space group | C2/c |
| Unit cell dimensions; Å, ° | a = 17.1131(2) |
|  | b = 6.72882(5) |
|  | c = 17.5783(2) |
|  | α= 90 |
|  | β= 111.3080(10) |
|  | γ = 90 |
| Volume; Å3 | 1885.79(4) |
| Z | 8 |
| Density (calculated); Mg/m3 | 1.935 |
| Absorption coefficient; mm-1 | 3.384 |
| F(000) | 1112 |
| Crystal size; mm | 0.308 x 0.261 x 0.168 |
| Theta range for data collection | 5.402 to 68.436°. |
| Index ranges | -20<=h<=14, -8<=k<=8, -21<=l<=21 |
| Reflections collected | 10660 |
| Independent reflections | 1720 [R(int) = 0.0153] |
| Completeness to theta = 67.684° | 100.0 % |
| Absorption correction | 3.384 mm-1 |
| Max. and min. transmission | 0.689 and 0.526 |
| Refinement method | Full-matrix least-squares on F2 |
| Data / restraints / parameters | 1720 / 0 / 142 |
| Goodness-of-fit on F2 | 1.155 |

**Table S2**. Selected bond lengths [Å] and angles [°] for cuglysala_

| Cu1-O1 | 1.9173(12) |
| --- | --- |
| Cu1-N1 | 1.9333(15) |
| Cu1-O2 | 1.9589(12) |
| Cu1-O1W | 2.0168(13) |
| O1-Cu1-N1 | 93.88(6) |
| O1-Cu1-O2 | 165.91(6) |
| N1-Cu1-O2 | 83.76(6) |
| O1-Cu1-O1W | 91.48(5) |
| N1-Cu1-O1W | 165.80(6) |
| O2-Cu1-O1W | 87.85(5) |
| C1-O1-Cu1 | 126.48(11) |
| C7-N1-Cu1 | 126.40(12) |
| C8-N1-Cu1 | 112.31(11) |
| C9-O2-Cu1 | 115.61(11) |
